# Enrollment, adherence and retention rates among musculoskeletal disorders rehabilitation practitioners in knowledge translation studies: a systematic review and meta-regression

**DOI:** 10.1186/s43058-024-00585-w

**Published:** 2024-05-03

**Authors:** D. Gaid, O. Eilayyan, S. Ahmed, A. Bussières

**Affiliations:** 1 School of Physical and Occupational Therapy, McGill University, Montreal, QC Canada; 2 Department of Physical Therapy, Al-Ahliyya Amman University, Amman, Jordan; 3 Centre de recherche interdisciplinaire en réadaptation du Montréal métropolitain (CRIR), Montreal, QC Canada; 4 The Research Institute of the McGill University Health Centre (RI-MUHC), Montreal, QC Canada; 5 Département Chiropratique, Université du Québec à Trois Rivières (UQTR), Trois-Rivières, QC Canada

**Keywords:** Systematic review, Rehabilitation, Meta-regression, Musculoskeletal disorders, Feasibility

## Abstract

**Background:**

Practitioners’ enrollment, adherence, and retention rates influence estimates of effectiveness in knowledge translation (KT) studies and remain important concerns for implementation researchers. This review aimed to systematically summarize the current evidence on feasibility measures as gauged by enrollment, adherence, and retention rates in KT evaluation studies targeting rehabilitation practitioners treating musculoskeletal disorders (MSDs).

**Methods:**

We searched five electronic databases from the inception to October 2022. We included KT studies that 1) had designs recommended by the Effective Practice and Organisation of Care, 2) targeted rehabilitation practitioners managing patients with MSDs, 3) delivered KT interventions according to the Expert Recommendations for Implementing Change classification, and 4) reported on the feasibility measures (e.g., enrollment, adherence, and retention). Descriptive statistics were conducted to report on study-, practitioners- and intervention-related factors influencing enrollment, adherence, and retention rates. Meta-regression weighted by the sample size of included studies was used to estimate the effect of factors on overall enrollment, adherence, and retention rates.

**Results:**

Findings from 33 KT studies reported weighted enrolment, adherence, and retention rate of 82% (range: 32%-100%), 74% (range: 44%-100%), and 65% (range: 36%-100%) respectively for both intervention and control groups. Factors positively influencing enrollment, adherence, and retention rates included designing short study period with short duration intervention.

**Conclusions:**

Intense (e.g., high frequency, short duration) single KT intervention was more appealing for practitioners. Future evaluation studies should clearly report follow-up data, and practitioners’ prior training, Results may not apply to non-MSD healthcare providers.

**Supplementary Information:**

The online version contains supplementary material available at 10.1186/s43058-024-00585-w.

Contributions to the literature

- Enrolment, adherence, and retention rates ranged between 65 and 82% across the KT studies.
- Single intense (e.g., high frequency, short duration) KT intervention was more appealing for practitioners.
- Interventions which require less effort and less commitment, and which save participants’ time have higher feasibility rates.

## Background

Musculoskeletal disorders (MSDs) are one of the most common health conditions experienced worldwide and are costly to the healthcare system [1], with one in six adults (15.6%) reporting chronic MSDs [2]. The most common chronic MSDs are osteoarthritis (OA), neck pain (NP), and low back pain (LBP). The World Health Organization estimates that 10% of individuals 60 years or older have significant clinical problems (e.g., functional limitation) that are attributed to OA [3]. Likewise, over 80% of the population experience LBP and NP during their lifetime [4–6]. Importantly, MSDs are associated with a high economic burden globally [7–9]. In 2010, the Public Health Agency of Canada highlighted that MSDs are associated with a higher economic burden than any other group of diseases, estimated at $37 billion [10].

Rehabilitation practitioners such as physiotherapists (PTs), occupational therapists (OTs), chiropractors (DCs) deliver care to over 11 million Canadians with MSDs, with estimates pointing to an increase to 15 million patients seeking care by 2031 [11]. Despite the availability of clinical practice guidelines to inform practice in rehabilitation [12–16], substantial research-practice gaps among rehabilitation clinicians persist [17–20]. The lack of adherence to recommended care can lead to negative effects on the health outcomes of individuals and communities and lead to inefficient use of limited health care resources [21, 22]. Knowledge Translation (KT) aims to promote the use of research evidence in healthcare systems [23]. Although, KT researchers evaluated the relative effectiveness of different KT interventions in changing healthcare professionals’ practice behaviour [24, 25], there is uncertainty regarding which KT interventions are likely to be effective in increasing the use of research findings [24, 26]. As KT interventions are multilevel interventions (e.g., professional, patient, or organizational level), other factors that may have impacted the success of the intervention [27, 28]. Low participant enrollment, adherence and retention rates (i.e., maintaining clinicians’ engagement throughout the course and up to the end of a trial) are major factors that can contribute to the success or failure of KT intervention [29] and influence the estimation of the effectiveness of any intervention [30] in healthcare research [31]. Difficulties in participants’ enrollment may lead to untimely delays in study initiation, financial burden, and failure to meet enrollment goals (i.e., underpowered trials) resulting in very expensive trials [31]. Thus, maximizing enrollment, adherence, and retention rates requires thoughtful planning, and specific strategies embedded in the trial process, and careful monitoring [31]. Assessing those rates may help researchers develop more appealing KT interventions that practitioners will more easily accept and sustain into their everyday practices, and improve the design of future trials, and consequently, increase their validity and generalizability [32]. Although successful enrollment and retention strategies have been described in clinical trials focusing on adults [33] and children [34], we are not aware of prior reviews having systematically assessed the enrollment, adherence, and retention rates in KT studies. This systematic review aimed to 1) estimate the enrollment, adherence, and retention rates of KT interventions targeting rehabilitation practitioners in charge of patients with MSDs, and 2) identify factors likely to impact on the enrollment, adherence, and retention rates.

## Methods

### Searches

A search strategy was developed in collaboration with a health-sciences librarian to ensure that we captured the maximum number of studies in rehabilitation sciences (Supplementary Material 1: Appendix 1). The search strategy was adapted from a previous review from this team [35], using subject headings (MeSH), keywords, and abstract/text words for MSDs, KT, and rehabilitation, and their synonyms. We searched published literature in scientific journals in the following five databases from the inception to October 2022: OVID MEDLINE, EMBASE, PsycINFO, CINAHL, and Cochrane databases, in English language. All identified citations were exported into EndNote after removing duplicates.

### Study inclusion and exclusion criteria

Three independent reviewers (DG, OE, KM) screened the titles and abstracts of studies identified by applying the eligibility criteria. The same reviewers then independently assessed full-text reports of potentially eligible studies. Reviewers met periodically to resolve disagreements and reach a consensus on the eligibility of studies at all stages.

#### Inclusion criteria

##### Study design

As recommended by the Effective Practice and Organisation of Care (EPOC) systematic reviews [36], we included the following study designs: Randomized Clinical Trials (RCTs), cluster randomized controlled trials, non-randomized controlled trials (NRCTs), or before-and-after studies.

##### Participants

All types of rehabilitation practitioners (e.g., PTs, OTs, Osteopaths, or DCs) managing patients with MSDs.

##### Intervention

KT interventions directed toward rehabilitation practitioners were selected according to the Expert Recommendations for Implementing Change (ERIC) classification [37], which provide comprehensive catalogue of KT interventions that can be used in isolation or combination in implementation research and practice (Supplementary Material 1: Appendix 2).

##### Outcomes

Three feasibility measures were considered: enrollment rate (defined as the proportion of participants who accepted to participate in the study over all eligible participants invited for the study, considering that the number of eligible practitioners was calculated after excluding individuals who did not meet the study’s inclusion criteria), adherence rate (defined as the proportion of participants who completed the intervention over all participants who were assigned to the intervention group), and retention rate (defined as the proportion of participants who completed through to the first follow-up point over the participants who started the study in each group “intervention group [IG] or control group [CG]”).

#### Exclusion criteria

Studies failing to report follow-up data for the participating practitioners were excluded. Studies published in abstract form, as conference proceedings, or protocol forms were also excluded.

### Data extraction strategy

A structured extraction sheet was created to collect and extract data from the eligible studies. We extracted data related to *study characteristics* (i.e., year of publication, country, study design, study duration, number of the study group, study duration, number of follow-up points, number of outcomes); *KT interventions* (i.e., type of KT intervention based on ERIC classification, number and duration of the KT interventions, mode of delivery, intensity of the intervention); *practitioners* (i.e., age, profession and types of MSDs managed; number of practitioners who were approached, eligible to participate, excluded, refused to participate, accepted to participate, and assigned to each study group “if applicable”; number of practitioners who adhered to the KT intervention, and who participated at least in the first follow-up point; and reasons for refusal to participate, and for dropping-out in each study group. The data were extracted by the principal investigator (DG) and reviewed by a second reviewer (OE).

### Study quality assessment

Quality assessment was not considered since the focus of this review was on enrollment, adherence, retention rates, and not the effectiveness and effect size of KT interventions likely to be impacted by criteria such as randomization process and missing data [38].

### Data synthesis and presentation

Statistical analyses were performed using the Statistical Analysis Systems (SAS version 9.3) [39] guided by two KT experts (AB and SA). Descriptive statistics were conducted to describe variables that possibly affected enrollment, adherence, and retention rates as proportion (%). Moreover, variables were categorized according to their nature and the frequency of data for *study characteristics* and *practitioners- and intervention*-related variables described above.

Meta-regression weighted by the sample size [38] of included studies was used to estimate the overall enrollment, adherence, and retention rates. Meta-regression was used since the outcome had a specific range (0–100%). The study, intervention, and practitioners-related variables aforementioned were used as the factors (i.e., predictors) of the enrollment, adherence, and retention rates. The overall enrollment and retention rates were calculated for intervention and control groups, and the overall adherence rate was estimated for intervention groups only. A meta-regression model was used to assess the correlation between the potential variables and enrollment, adherence, and retention rates. These three rates were treated as continuous variables. A *p*-value of less than 0.05 was considered as statistically significant.

## Results

### Descriptive statistics

**Fig. 1:**
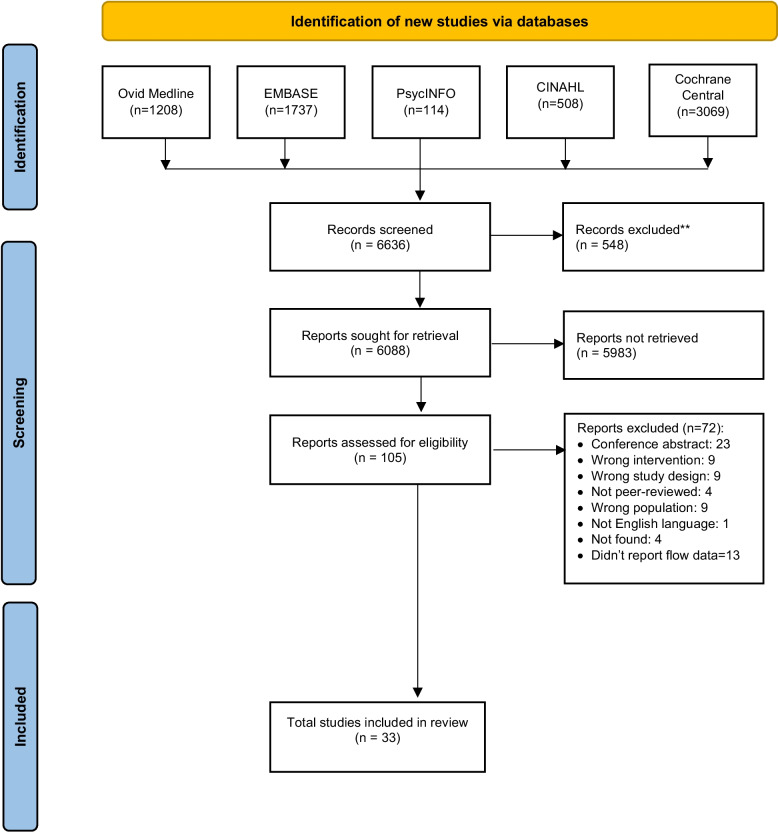
PRISMA flow diagram representing the process of study selection

The search strategy yielded 6088 records after duplicate removal. Screening for titles and abstracts identified 105 potentially eligible articles of which 33 studies met our inclusion criteria [40–69] (See Fig. 1. PRISMA flowchart). Table 1 provides a description of the study- practitioners- and KT intervention characteristics of the included studies.

**Table 1:** Characteristics of the included studies

Author Name	Year	Country	Design	No. of study group	No. of intervention group	No. of control group	Study duration (months)	No. Follow-ups	No. of outcomes	Practitioners population	Patient population
Ammendolia et al.	2004	Canada	Quasi-experimental	2	19	13	15	1	2	DCs	LBP
Stevenson et al.	2004	UK	Cluster RCT	2	1	13	6	2	2	PTs	MSDs
Bekkering et al.	2005	Netherlands	Cluster RCT	2	52	61	19	1	1	PTs	LBP
Rebbeck et al.	2006	Australia	Cluster RCT	2	14	13	12	1	3	PTs	NP
Gross et al.	2009	Canada	Before-after	1	241	N/A	12	1	2	PTs	MSDs
Overmeer et al.	2009	UK	Before-after	1	22	N/A	6	1	4	PTs	MSDs
Joshua A Cleland et al.	2009	USA	RCT	2	10	9	26	1	0	PTs	NP
Bussières et al.	2010	Switzerland	RCT	2	80	40	4	2	1	DCs	MSDs
Evans et al.	2010	UK	RCT	2	876	882	6	1	2	PTs, DCs, Dos	LBP
Fruth et al.	2010	USA	Before-after	1	24	N/A	3	2	1	PTs	LBP
Demmelmaier et al.	2010	Sweden	Before-after	1	4	N/A	9	6	5	PTs	BP
Peter et al.	2013	Netherlands	RCT	2	108	95	3	2	3	PTs	OA
Rebbeck et al.	2013	Australia	Before-after	1	93	N/A	9	1	3	PTs, DCs, Dos	NP
Bernhardsson et al.	2014	Sweden	Non-RCT	2	277	171	12	1	3	PTs	MSDs
Buchanan, Helen et al.	2014	South Africa	Pragmatic RCT	2	28	28	3	1	2	OTs	EPB
Dulmen et al.	2014	Netherlands	Cluster RCT	2	49	41	6	1	3	PTs	LBP
Beneciuk et al.	2015	USA	RCT	2	6	6	8	2	1	PTs	LBP
Maas et al.	2015	Netherlands	RCT	2	73	76	6	1	4	PTs	UL
Peter et al.	2015	Netherlands	RCT	2	133	151	3	2	4	PTs	OA
Karvonen et al.	2015	Finland	Before-after	1	6	N/A	2	1	1	PTs	LBP
Chipchase et al.	2016	Australia	Single-blind RCT	2	12	11	2	1	1	PTs	NP
Käll et al.	2016	Sweden	Prospective CT	2	277	171	12	1	3	PTs	MSDs
Tilson et al.	2016	USA	Before-after	1	1102	N/A	12	2	6	PTs	LBP
Richmond et al.	2016	UK	RCT	2	16	19	6	1	7	PTs	LBP
Dhopte et al.	2019	Canada	Cluster RCT	2	27	20	3	1	2	DCs	NP
Carlfjord et al.	2019	Sweden	Before-after	1	261	N/A	12	2	2	PTs	NP
Hurley et al.	2019	Ireland	Before-after	1	7	N/A	1.5	1	3	PTs	MSDs
Williamson et al.	2020	UK	Implementation hybrid	1	584	N/A	18	2	7	PTs and OTs	MSDs
Schröder et al.	2020	Sweden	Before-after	1	116	N/A	12	3	3	PTs	LBP
Sugavanam et al.	2020	UK	Before-after	1	1324	N/A	12	3	2	PTs, OTs, DCs	LBP
Draper-Rodi et al.	2021	UK	Cross-sectional stepped-wedge pilot	2	23	22	1.5	1	2	Osteopath	LBP
Longtin et al.	2021	Canada	One-arm prospective feasibility	1	24	N/A	1.5	2	2	PTs	LBP
Eilayyan et al.	2022	Canada	Mixed methods pilot clustered-clinical	2	7	9	24	3	4	DCs	BP

*RCT* Randomized Controlled Trials, *CT* Controlled Trials, *DCs* Chiropractors, *PTs* Physiotherapists, *OTs* Occupational Therapists, *LBP* Low Back Pain, *MSDs* Musculoskeletal disorders, *NP* Neck Pain, *BP* Back Pain, *OA* Osteoarthritis, *EPB* Evidence Based Practice, *UL* Upper Limb

#### Studies’ characteristics

The included studies were published between 2004 and 2022 in Europe (61%, *n* = 20), North America (27%, *n* = 9), Australia (9%, *n* = 3) or elsewhere (3%, *n* = 1). Most of the studies (61%, *n* = 20) were controlled trials, such as RCT (*n* = 8) [40, 43, 46, 49, 53, 55, 56, 68], cluster-RCT (*n* = 5) [42, 58, 59, 61, 67], pragmatic RCT (*n* = 1) [45], prospective controlled trials (CT) (*n* = 1) [52], single-blind RCT (*n* = 1) [47], and non-RCT (*n* = 1) [44], mixed methods trial (*n* = 1) [70, 71], and before-and-after control study (*n* = 1) [41]. The remaining studies were before-and-after studies (39%, *n* = 13) [48, 50, 51, 54, 57, 60, 62–66, 69, 72]. Study duration ranged from 2 to 26 months. The median of the follow-up points was one follow-up. More than half (57%, *n* = 19) [40–42, 44, 45, 47, 49, 51–54, 57, 58, 61, 65, 67–69, 71] of the included studies had one follow-up point or two follow-up points (30%, *n* = 10) [43, 46, 50, 55, 56, 59, 60, 64, 66, 72], while few studies had more than two follow-up points (12%, *n* = 4) [48, 62, 63, 70].

#### Practitioners’ characteristics

The practitioners’ mean age was provided in 21 studies [40–43, 45, 47–50, 53–57, 60–62, 65, 67, 70, 72]; ranging from 28 to 47.5 years for intervention groups (x̅ = 40 years; SD = 5.1) and 33 to 54.3 years for control groups (x̅ = 43 years; SD = 4.7). Most of the studies targeted PTs (70%, *n* = 23) [40, 42–44, 47, 48, 50–56, 58–62, 65, 66, 68, 69, 72]. The remaining studies targeted a mixed types of practitioners (PTs, OTs, DCs) (13%, *n* = 4) [49, 57, 63, 64], DCs (12%, *n* = 4) [41, 46, 67, 70], OTs (3%, *n* = 1) [45], or osteopaths (3%, *n* = 1) [71]. The types of disorders includes back pain (45%, *n* = 15) [41–43, 48–50, 60–63, 65, 68, 70–72], MSKs in general (27%, *n* = 9) [44–46, 51–54, 59, 69], NP (18%, *n* = 6) [40, 47, 57, 58, 66, 67], and OA (6%, *n* = 2) [55, 56], and rheumatoid arthritis (3%, *n* = 1) [64].

#### KT interventions’ characteristics

Based on the ERIC classification [37], the predominant type of KT interventions were educational meetings (97%, *n* = 32) [40–48, 50–72], distribution of educational materials (58%, *n* = 19) [40–45, 48, 49, 51, 52, 54–56, 58, 60, 61, 63, 64, 70], audit and feedback (30%, *n* = 10) [40, 42, 45, 48, 53, 54, 56, 60–62], local opinion leaders (24%, *n* = 8) [41, 51, 56–59, 62, 70], reminders (21%, *n* = 7) [44–46, 52, 53, 62, 70], facilitation (15%, *n* = 5) [40, 47, 55, 61, 68], educational outreach visits (12%, *n* = 4) [40, 41, 58, 62], ongoing consultation (12%, *n* = 4) [40, 44, 52, 69], and developing centralize technical assistance (12%, *n* = 4) [44, 52, 60, 72]. Developing educational materials [44, 64, 69], and creating a learning collaborative [45, 60, 69] were used in equal frequency (9%, *n* = 3). Providing clinical supervision [40, 60] and intervening with patients to enhance adherence [44, 64] (6%, *n* = 2), preparing patients to be active participants [52], local consensus discussions [60], conducting ongoing training [69], and using mass media [41] (3%, *n* = 1) were uncommon (Table 2). An equal proportion of studies combined two to three interventions (42%, *n* = 14) [42, 43, 46–48, 51, 53–55, 57, 59, 63, 68, 72] or more than three interventions (39%, *n* = 13) [40, 41, 44, 45, 52, 56, 58, 60–62, 64, 69, 70]. Few studies employed a single intervention (18%, *n* = 6) [49, 50, 65–67, 71]. The majority of the studies delivered the KT intervention in person (76%, *n* = 25) [40–48, 50–62, 65, 66, 72] or online (21%, *n* = 7) [63, 64, 67–71]. Only one study used postal dissemination (3%, *n* = 1) [49].

**Table 2:** Types of KT intervention classified according to ERIC classification

Author Name	EM	DEM	AF	LOL	RD	FT	OV	OC	CTA	DvEM	CLC	PCS	IPEA	PPAP	LCD	COT	MM
Ammendolia et al. 2004 [41]	√	√		√			√										√
Stevenson et al. 2004 [59]	√			√													
Bekkering et al. 2005 [42]	√	√	√														
Rebbeck et al. 2006 [58]	√	√		√			√										
Gross et al. 2009 [51]	√	√		√													
Overmeer et al. 2009 [54]	√	√	√														
Joshua A Cleland et al. 2009 [40]	√	√	√			√	√	√				√					
Bussières et al. 2010 [46]	√				√												
Evans et al. 2010 [49]		√															
Fruth et al. 2010 [50]	√																
Demmelmaier et al. 2012 [73]	√	√	√														
Peter et al. 2013 [56]	√	√	√	√													
Rebbeck et al. 2013 [25, 57]	√			√													
Bernhardsson et al. 2014 [44]	√	√			√			√	√	√			√				
Buchanan, Helen et al. 2014 [45]	√	√	√		√						√						
Dulmen et al. 2014 [61]	√	√	√			√											
Beneciuk et al. 2015 [43]	√	√															
Maas et al. 2015 [53]	√		√		√												
Peter et al. 2015 [55]	√	√				√											
Karvonen et al. 2015 [65]	√																
Chipchase et al. 2016 [47]	√					√											
Käll et al. 2016 [52]	√	√			√			√	√					√			
Tilson et al. 2016 [60]	√	√	√						√		√	√			√		
Richmond et al. 2016 [68]	√					√											
Dhopte et al. 2019 [67]	√																
Carlfjord et al. 2019 [66]	√																
Hurley et al. 2019 [69]	√							√		√	√					√	
Williamson et al. 2020 [64]	√	√								√			√				
Schröder et al. 2020 [62]	√		√	√	√		√										
Sugavanam et al. 2020 [63]	√	√															
Draper-Rodi et al. 2021 [71]	√																
Longtin et al. 2021 [72]	√								√								
Eilayyan et al. 2022 [70]	√	√		√	√												

*EM* Educational Meetings, *DEM* Distribution of Educational Materials, *AF* Audit and Feedback, *LOL* Local Opinion Leaders, *RD* Reminders, *FT* Facilitation, *OV* Outreach Visits, *OC* Ongoing Consultation, *CTA* Centralize Technical Assistance, *DvEM* Developing Educational Materials, *CLC* Creating a Learning Collaborative, *PCS* Providing Clinical Supervision, *IPEA* Intervening with Patients to Enhance Adherence, *PPAP* Preparing Patients to be Active Participants, *LCD* Local consensus discussions, *COT* Conducting Ongoing Training, *MM* Mass media

The other groups (named as control groups) either received no intervention (*n* = 6) [41, 44, 49, 52, 55, 71], or educational interventions (*n* = 5) (e.g., external coach [61], opinion leader, audit and feedback [56], practicing skills [45], peer assessment approach [53], and outreach visit, clinical supervision, ongoing consultation [40]), educational materials only (*n* = 4) [42, 58, 67, 70], less frequent educational sessions (*n* = 3) [43, 47, 68], or a similar intervention but on different topics (*n* = 2) [46, 59].

### Enrollment, adherence, and retention data

**Fig. 2:**
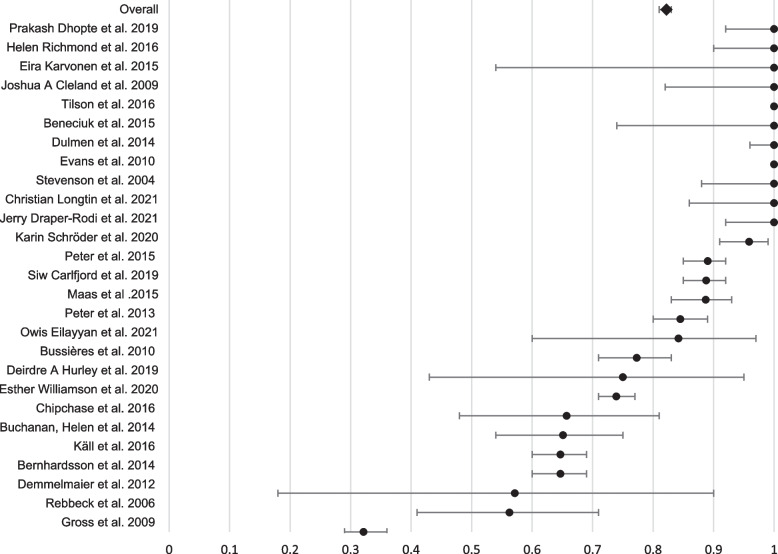
Forest plot of the enrollment rates

**Fig. 3:**
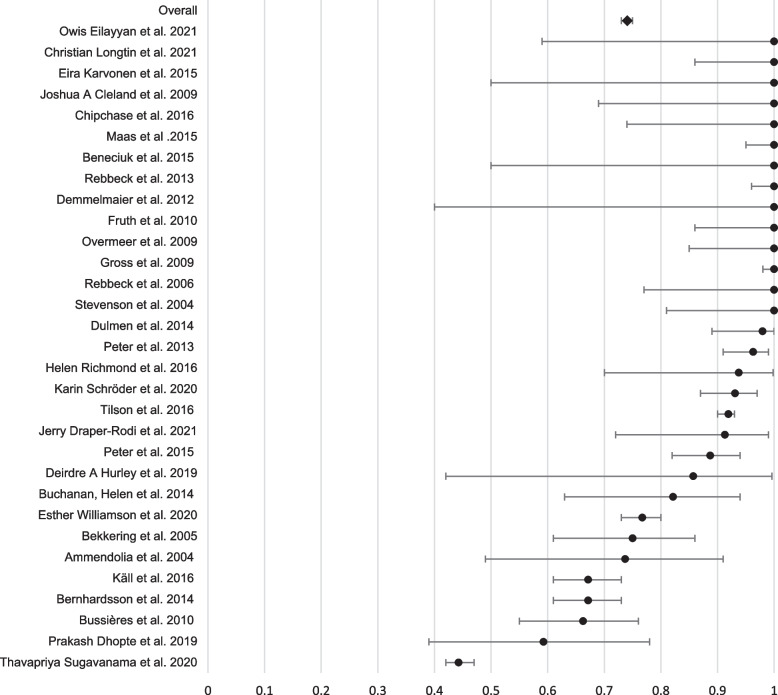
Forest plot of the adherence rates

**Fig. 4:**
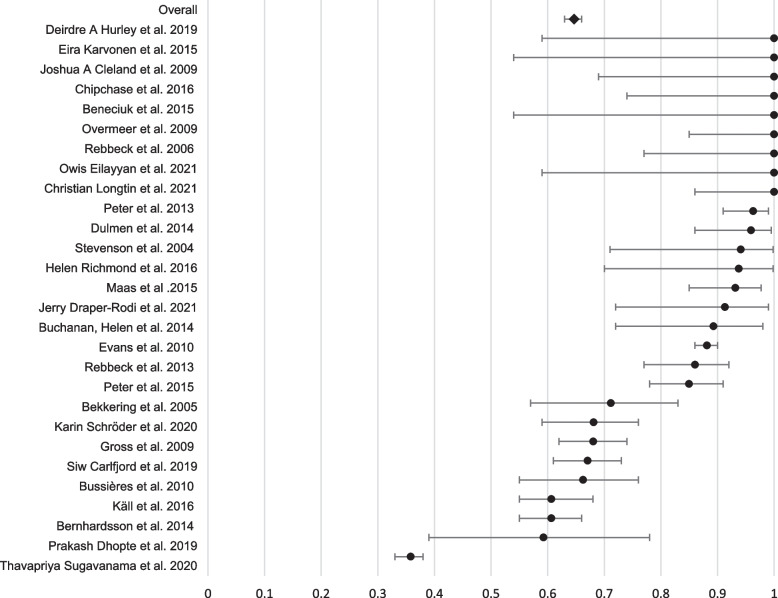
Forest plot of the retention rates

1. Enrollment rate: Overall, 7146 eligible practitioners from 27 KT studies were invited to participate, of whom 5880 agreed to participate [40, 43–49, 51–53, 55, 56, 58–62, 64–68, 70–72]. The overall unweighted enrollment rate was 84% and the weighted enrollment rate was 82%, ranging from 32% [51] to 100% in eleven studies [40, 43, 49, 59–61, 65, 67, 68, 71, 72], including 8 controlled trials (CTs) [40, 43, 49, 59, 61, 67, 68, 71] and 3 before-and-after studies [60, 65, 72].
2. Adherence rate: The included studies reported the number of practitioners who participated in the educational meetings only, not for every type of KT interventions separately. Thus, the adherence rate was calculated for educational meetings only, however, the calculated adherence rate can well exemplify the adherence rate for the other associated KT interventions in each study as educational meetings were mostly delivered concurrently with other intervention such as distribution of education materials, local opinion leaders, facilitation, audit and feedback, etc. Overall, 4775 practitioners were assigned to attend the educational meetings; 3537 did in fact attend as per protocol, with an overall unweighted adherence rate of 88% and a weighted adherence rate of 74% (73% for before-and-after studies [48, 51, 60, 62–66, 69, 72] and 78% for controlled trials [40, 43–47, 49, 52, 53, 55, 56, 58, 59, 61, 67, 68, 70, 71]). A 100% adherence was reached in 14 studies [40, 43, 47, 48, 50, 51, 53, 54, 57–59, 65, 70, 72], of which half were controlled trials [40, 43, 47, 53, 58, 59, 70]. The length of educational meetings ranged from 1 to 8 h. However, the lowest adherence (44%) was observed in a before-and-after study targeting individuals with LBP [63]. The educational sessions was available for online browsing for up to 3 weeks.
3. Retention rate: The number of practitioners who completed the first follow-up point was reported in 28 studies (before-and-after studies [*n* = 9] [51, 54, 57, 62, 63, 65, 66, 69, 72] and controlled trials [*n* = 19] [40, 42–47, 49, 52, 53, 55, 56, 58, 59, 61, 67, 68, 70, 71]). For before-and after studies, 1031 practitioners out of 2094 completed the first follow-up point, with a retention rate of 49% (range: 36% to 100%). For controlled trials, the retention rate was 80% for interventions groups (1672 / 2085) and 81% for control groups (1494 / 1838), with an overall rate of 81% for both groups. The overall unweighted retention rate across all studies was 85% and the weighted retention rate was 65%, ranging from 36% in a before-and-after study targeting mixed types of practitioners [63] to 100% in 7 studies, including 3 CTs [40, 43, 47] and 4 before-and-after studies [54, 65, 69, 72]. Figures 2, 3, and 4 present forest plots for the enrollment, adherence, and retention rates.

### Reasons for refusal to participate

Only 8 studies [42, 45, 48, 55, 56, 62, 67–69] (27%) reported on the reasons for refusing to participate, namely lack of time (*n* = 4) [45, 48, 56, 68], lack of interest (*n* = 6) [42, 45, 48, 56, 62, 67], having a health condition preventing them from participating (*n* = 3) [45, 55, 67], or unavailability during the study time (*n* = 2) [56, 69]. Other reasons reported only once were distance from the intervention site [56], holidays [56], other priorities [56], work obligations [68], work schedule conflict [55, 68], moved out of country [67], fail to submit consent [45], retired [67], joining politics [67], and invalid address [67].

### Reasons for drop outs

Thirteen studies [42, 44, 45, 52, 53, 56, 57, 59, 61, 62, 67–69] (43%) reported the reasons for participants’ drop out throughout the course of the studies after consenting to participate, including: personal life changes (*n* = 9) (i.e., change of job, retirement, maternal leave or pregnancy) [42, 45, 53, 56, 57, 59, 62, 67, 68], lack of time (*n* = 5) [42, 53, 56, 57, 67], unknown reason (*n* = 3) [42, 56, 61], work conflict (*n* = 3) [45, 56, 69], unable to contact the participant (*n* = 3) [44, 52, 57], lack of interest (*n* = 2) [42, 45], lack of compliance (*n* = 2) [62, 69], being out of town (*n* = 1) [67], and transportation problem (*n* = 1) [45].

### Factors influencing enrollment, adherence, and retention rates

In general, the meta-regression showed that all the aforementioned factors (i.e., variables related to studies, interventions, and practitioners) significantly affected the rates of enrollment, adherence, and retention. All comparisons were significant at *P*-value < 0.0001 (Table 3).

1. Factors influencing enrollment rate: For study-related factors, the enrollment rate was 12% higher in Europe compared to North America. Further, before-and-after studies had about 12% lower enrollment rate compared with controlled trials; implementing a study with more than one study group was associated with a 12% higher enrollment rate, while having more than one follow-up point was associated with an 11% greater enrollment rate. For practitioners-related factors, enrollment rate was higher for mixed types of MSDs practitioners by 15% compared to delivering intervention to single type of practitioners (e.g., OTs, PTs, DCs). Also, enrollment rate was higher for practitioners managing BP and NP by 33% and 19%, respectively, compared to practitioners who manage mixed type of MSDs. As for KT intervention-related factors, enrolment rate was lower when employing two to three interventions, or more than three interventions (38% and 16%) higher respectively compared to employing single intervention; but 16% higher when delivering KT intervention online (virtually) compared to in-person; 19% higher when delivering educational meeting for more than 4 h; and 29% higher when conducting the educational meetings more than one time.
2. Factors influencing adherence rate: Interestingly, adherence rate for study-related factors was 28% lower in Europe when compared to North America, 13% lower when implementing a study for over 6 months; 11% lower when there was more than one follow-up point; but 27% higher when measuring more than two professional outcomes. For the practitioners-related factors, adherence rate was lower when recruiting mixed types of practitioners, DCs and DOs, OTs, compared to PTs by 32%, 17%, and 5%, respectively. Also, adherence rate was higher for practitioners managing NP by 13%, and lower by 11% for practitioners managing BP, both compared to practitioners who manage mixed type of MSDs. As for the KT intervention-related factors, adherence rate was 22% lower when employing two to three interventions; 32% lower when delivering KT intervention online compared to in-person mode; but 16% greater when implementing a meeting length more than 4 h; 12% higher when conducting the educational meetings more than one time; and 29% higher when delivering the KT intervention for a long period (e.g., 1 month up to 6 months).
3. Factors influencing retention rate: Similarly for study-related factors, retention rate was 17% higher in other countries (i.e., Australia) compared to North America. Retention rate was 31% lower when in before-and-after study compared to controlled trials; 22% lower when implementing a study for over 6 months; but 31% greater when implementing a study in more than one study group. The retention rate was also 28% lower when having more than one follow-up point; and 14% higher when measuring more than two professional outcomes. Concerning the practitioners-related factors, retention rate was 16% higher when recruiting OTs, but lower 15% when recruiting mixed types of MSKs practitioners compared to recruiting PTs. Also, retention rate was lower for practitioners managing BP by 12% compared to practitioners who manage mixed type of MSDs. As for the KT intervention-related factors, retention rate was 31% and 13% lower when employing two to three interventions or more than three interventions vs employing a single intervention, respectively. Compared to in-person mode of delivery, retention rate was also 35% lower for online KT interventions, but 15% higher for postal dissemination. Retention rate was 13% greater when conducting the educational meetings more than one time and 29% higher when delivering the KT intervention for a long period (e.g., 1 month up to 6 months).

**Table 3:** Meta-regression of factors influencing rates of enrollment, adherence and retention

**Category**	**Variable**	**Sub-categories**	**Enrollment**		**Adherence**		**Retention**	
			**Beta-coefficient (95% CI)**	***P*-value**	**Beta-coefficient (95% CI)**	***P*-value**	**Beta-coefficient (95% CI)**	***P*-value**
Factors Related to the Study	Year of publication	Before 2011 (ref)	-	-	-	-	-	-
		After 2011	0.047 (0.036-0.059)	< .0001	-0.18 (-0.2- -0.17)	< .0001	-0.27 (-0.26- -0.25)	< .0001
	Country	North America (ref)	-	-	-	-	-	-
		Europe	0.12 (0.11–0.14)	< .0001	-0.286 (-0.29- --0.278)	< .0001	-0.09 (-0.1- -0.07)	< .0001
		Others	-0.1133	< .0001	0.037 (0.025-0.049)	< .0001	0.17 (0.15-0.19)	< .0001
	Study design	CTs (ref)	-	-	-	-	-	-
		Pre-Post	-0.12 (-13- -0.11)	< .0001	-0.057 (-0.068- -0.046)	< .0001	-0.31 (-0.32- -0.3)	< .0001
	Study duration	Equal/less than 6 months (ref)	-	-	-	-	-	-
		More than 6 months	-0.02 (-0.028- -0.012)	0.0355	-0.13 (-0.15- -0.12)	< .0001	-0.23 (-0.24- -0.21)	< .0001
	Number of study groups	One group (ref)	-	-	-	-	-	-
		More than one group	0.12 (0.11-0.13)	< .0001	0.057 (0.046-0.068)	< .0001	0.31 (0.30-0.32)	< .0001
	Number of follow-up points	One follow up point (ref)	-	-	-	-	-	-
		More than one follow up point	0.11 (0.1–0.12)	< .0001	-0.11(-0.12- -0.099)	< .0001	-0.28 (-0.29- -0.27)	< .0001
	Number of practitioners’ outcomes measures	Equal/less than two outcomes (ref)	-	-	-	-	-	-
		More than 2 outcomes	0.025 (0.014-0.035)	< .0001	0.27 (0.26-0.28)	< .0001	0.14 (0.13-0.15)	< .0001
Factors Related to the practitioners	Practitioners’ profession	PTs (ref)	-	-	-	-	-	-
		OTs	-0.115 (-0.12- -0.11)	< .0001	-0.057 (-0.063- -0.54)	< .0001	0.164 (0.157-0.17)	< .0001
		DCs and DOs	0.08 (0.06–0.09)	< .0001	-0.17 (-0.19- -0.15)	< .0001	-0.02 (-0.04-0.0005)	0.055
		Mix practitioners	0.15 (0.14-0.16)	< .0001	-0.32 (-0.33- -0.31)	< .0001	-0.15 (-0.16- -0.14)	< .0001
	Managing MSDs disorders	MSDs (ref)	-	-	-	-	-	-
		BP	0.338 (0.33-0.344)	< .0001	-0.11 (-0.12- -0.1)	< .0001	-0.12 (-0.14- -0.11)	< .0001
		NP	0.19 (0.18-0.21)	< .0001	0.13 (0.1-0.15)	< .0001	0.017 (0.004-0.03	0.0118
Factors Related to the KT intervention	Number of KT intervention	One intervention (ref)	-	-	-	-	-	-
		From two to three interventions	-0.38 (-0.39- -0.36)	< .0001	-0.22(-0.26- -0.18)	< .0001	-0.31 (-0.32- -0.30)	< .0001
		More than three intervention	-0.16 (-168- -0.158)	< .0001	-0.002 (-0.05- 0.04)	0.9205	-0.126 (-0.137- --0.114)	< .0001
	Duration of the KT intervention	1 day up to 3 weeks (ref)	-	-	-	-	-	-
		1 month up to 6 months	0.032 (0.026-0.037)	< .0001	0.29 (0.276-0.298)	< .0001	0.29 (0.28-0.30)	< .0001
	Method of intervention delivery	In-person (ref)	-	-	-	-	-	-
		Online	0.16 (0.15-0.17)	< .0001	-0.32 (-0.33- -0.31)	< .0001	-0.35 (-0.36- -0.34)	< .0001
		Postal dissemination	/	/	/	/	0.149 (0.143-0.155)	< .0001
	Length of educational meetings	Equal/less than 4 h (ref)	-	-	-	-	-	-
		More than 4 h	0.19 (0.186-0.198)	< .0001	0.156 (0.151-0.162)	< .0001	0.03 (0.02-0.04)	< .0001
	Frequency of educational meetings	Once (ref)	-	-	-	-	-	-
		Repetitive	0.29 (0.28-0.3)	< .0001	0.125 (0.12-0.13)	< .0001	0.13 (0.12-0.14)	< .0001

*CI* Confidence Interval, *CTs* Controlled Trials, *DCs* Chiropractors, *PTs* Physiotherapists, *OTs* Occupational Therapists, *ref* Reference Category, / Missing or Not Applicable

## Discussion

To our knowledge, this is the first review estimating the enrolment, adherence, and retention rates of KT interventions targeting rehabilitation practitioners managing patients with MSDs. Results of the current review showed high enrolment rate (82%) in KT studies, and relatively high adherence (74%) and retention rates (65%) across studies.

This review supported that designing a study with more than one group of practitioners with a controlled arm is associated with higher feasibility rates. Similar findings were reported by Lixin Song et al. [74] when examining the enrollment and retention rates clinical trials of patients with cancer and their caregivers. Studies of shorter period (less than 6 months) with only one follow-up point with multiple outcome measures were associated with higher feasibility rates. These findings are possibly explained by the difficulties for practitioners to commit to their regular work schedule over a long period, thereby limiting their ability to report outcomes over multiple follow-up points.

We uncovered a number of appealing intervention-related features for rehabilitation practitioners that seems to promote all three feasibility measures. First, employing a single intervention for a short period of time (1 month up to 6 months) is significantly associated with the higher rates. Systematic reviews of KT studies have suggested that single active KT interventions may be as effective as multi-component intervention in changing practice [26, 75–77]. The complexity of interventions may dampen the key messages and diminish the ability of practitioners to digest the presented information [78]. Previous studies reported a higher enrollment rate when recruiting participants for studies with ≥ 4 months intervention duration [74, 79]. Second, implementing a long educational meeting (more than 4 h) for more than one time is associated with higher rates; this possibly means that practitioners prefer for instance to concentrate on a full day workshop offered multiple times (i.e., long-term engagement), instead of having several short meetings during their busy working day when being exposed to the content of the KT intervention. Lastly, delivering KT intervention virtually or in-person mode provided mixed results with virtual mode being associated with a higher enrolment rate, whereas in-person mode was associated with greater adherence and retention rates; these findings support that online interventions could be considered as time and effort saving modes of delivery. Feasibility rates don’t seem to be harmonically affected by the practitioners’ profession or the type of MSDs they manage. Considering those intervention-related factors may secure higher practitioners’ involvement in the KT studies for longer duration.

## Strengths and limitations

This systematic review followed rigorous methodology, including a comprehensive search strategy developed in collaboration with a medical science librarian, the use of multiple electronic databases. However, this review is not without limitations. First, several studies failed to report on the number of practitioners who were eligible to participate in the study. Second, other variables that could be influential, such as practitioners’ educational backgrounds and practitioners’ beliefs in KT interventions, could not be included in the analyses as sufficient information on these variables was not available. Third, assessing the impact of each type of KT interventions separately on the feasibility rates was not possible due to the overlapping of the KT interventions in each study. Fourth, the included studies fail to reported the number of participants received each KT intervention separately. Finally, this review was restricted to KT interventions targeting MSDs rehabilitation practitioners only. Thus results may not apply to other healthcare disciplines.

## Conclusion

This systematic review identified 33 studies employing KT interventions to promote the uptake of research evidence by MSDs rehabilitation practitioners, including PTs, OTs, DCs, and osteopaths. Findings showed that enrolment, adherence, and retention rates ranged between 65 and 82% across the KT studies. Moreover, this review showed that single intense (e.g., high frequency, short duration) KT intervention was more appealing for practitioners. Interventions which require less effort and less commitment, and which save participants’ time have higher feasibility rates. KT researchers should consider the time required from healthcare practitioners to participate in a KT studies to maximize the feasibility rates, and consequently increase the generalizability of their findings.

### Supplementary Information

**Supplementary Material 1.**

## Data Availability

N/A

## Abbreviations

CTs: Controlled Trials
DCs: Chiropractors
EPOC: Effective Practice and Organisation of Care
ERIC: Expert Recommendations for Implementing Change
KT: Knowledge Translation
LBP: Low back pain
MSDs: Musculoskeletal Disorders
NP: Neck pain
NRCTs: Non-Randomized Controlled Trials
OA: Osteoarthritis
OTs: Occupational Therapists
PTs: Physiotherapists
RCTs: Randomized Clinical Trials
