# Supplementary material for: Enrollment, adherence and retention rates among musculoskeletal disorders rehabilitation practitioners in knowledge translation studies: a systematic review and meta-regression
Source: Implement Sci Commun. 2024 May 3;5:51. doi: 10.1186/s43058-024-00585-w (PMC11069130; doi:10.1186/s43058-024-00585-w)
Supplement: Supplementary file 1 — Supplementary Material 1. [file 43058_2024_585_MOESM1_ESM.docx]

**Appendix (1): Search strategy in Medline, Embase, PsycInfo, CINAHL and Cochrane Central**

**1. Ovid MEDLINE:** **Ovid MEDLINE(R) ALL 1946 to January 28, 2021**

| 1 | exp Translational Medical Research/ or "Diffusion of Innovation"/ or Information Dissemination/ or Health Knowledge, Attitudes, Practice/ |
| --- | --- |
| 2 | exp Practice Guidelines as Topic/ or Practice Guideline/ or Continuing education/ |
| 3 | (KT adj1 (intervention or interventions or plan or plans or policy or policies or strategy or strategies)).mp. |
| 4 | ((knowledge or research or information or evidence or science or finding*) adj1 (translation or transfer or exchange or action or practice or decision or implementation or management or dissemination or application or share or sharing or uptake or utili?ation or mobile?ation or integration or communication or adoption or diffusion or brokering or creation)).mp. |
| 5 | (knowledge to action or research to practice or diffusion of innovations or scale up or translational research or translation of research findings or implementation or continuing education or organi*ational innovation or complex intervention or behavio*r change intervention* or "technology transfer").mp. |
| 6 | ((Continuing adj2 professional adj2 development) or (implementation adj2 science)).mp. |
| 7 | (intervention? adj2 (complex or education or multifacet* or multi-facet* or organi?ation* or tailor* or target* or interdisciplin* or multi-disciplin* or multidiscipline* or evidence-based or evidence-driven)).mp. |
| 8 | 1 or 2 or 3 or 4 or 5 or 6 or 7 |
| 9 | Physical Therapy Modalities/ |
| 10 | "Physical Therapy (Specialty)"/ |
| 11 | Cardiac rehabilitation.mp. or Cardiac Rehabilitation/ |
| 12 | Lung Diseases, Obstructive/ or Pulmonary Disease, Chronic Obstructive/ or pulmonary rehabilitation.mp. |
| 13 | neurological rehabilitation.mp. or Neurological Rehabilitation/ or Stroke Rehabilitation/ |
| 14 | Exercise Therapy.mp. or exp Exercise Therapy/ |
| 15 | Manipulation, Chiropractic/ or Chiropractic/ |
| 16 | Mobilization.mp. |
| 17 | exp Osteopathic Medicine/ |
| 18 | Osteopathic.mp. or Manipulation, Osteopathic/ or Osteopathic Physicians/ or Osteopathic Medicine/ |
| 19 | "Physical and Rehabilitation Medicine"/ |
| 20 | (physical therap* or physiotherap*).mp. |
| 21 | Occupational therapy/ or occupational therap*.mp. |
| 22 | Chiropract*.tw. |
| 23 | Chiroprax*.tw. |
| 24 | Chiropractic.mp. |
| 25 | OMT.mp. |
| 26 | Osteopath*.ti,ab. |
| 27 | (Osteopath* adj1 manipulat$).mp. |
| 28 | Manual therap*.mp. |
| 29 | Sport* therap*.mp. |
| 30 | Sport physician*.mp. |
| 31 | Sports medicine/ |
| 32 | Massage therap*.mp. |
| 33 | kinesiolog*.mp. |
| 34 | Physiatr*.mp. |
| 35 | Physical medicine.mp. |
| 36 | (physical adj2 medicine).mp. |
| 37 | Rehabilitation medicine.mp. |
| 38 | Orthoped*.mp. |
| 39 | Orthopaed*.mp. |
| 40 | Orthopod*.mp. |
| 41 | Doctor of Podiatric Medicine.mp. |
| 42 | Podiatr*.mp. |
| 43 | Chiropod*.mp. |
| 44 | Hand therap*.mp. |
| 45 | Foot therap*.mp. |
| 46 | 9 or 10 or 11 or 12 r 13 or 14 or 15 or 16 or 17 or 18 or 19 or 20 or 21 or 22 or 23 or 24 or 25 or 26 or 27 or 28 or 29 or 30 or 31 or 32 or 33 or 34 or 35 or 36 or 37 or 38 or 39 or 40 or 41 or 42 or 43 or 44 or 45 |
| 47 | Musculoskeletal Disease.mp. or Musculoskeletal Diseases/ |
| 48 | Musculoskeletal adj2 (condition or abnorm$ or disorder or defect).mp. |
| 49 | exp back pain/ |
| 50 | exp neck pain/ |
| 51 | exp Shoulder pain/ |
| 52 | exp Tennis Elbow/ |
| 53 | exp Tendinopathy/ |
| 54 | exp Whiplash Injuries/ |
| 55 | exp Sciatica/ |
| 56 | exp Joint Diseases/ |
| 57 | exp Spinal Diseases/ |
| 58 | exp Joint dislocation/ |
| 59 | exp Joint instability/ |
| 60 | exp Spondylarthritis/ |
| 61 | exp osteoarthritis/ |
| 62 | exp Osteoporosis/ |
| 63 | exp cervical vertebrae/ or intervertebral disk/ or lumbar vertebrae/ or thoracic vertebrae/ |
| 64 | exp Back/ |
| 65 | exp Spine/ |
| 66 | exp Intervertebral Disk Displacement/ |
| 67 | (pain adj1 (neck or back or shoulder? or elbow? or forearm? or wrist? or hand? or arm? or hip? or knee? or ankle? or leg? or foot or feet)).tw. |
| 68 | (epicondylitis or tendonitis or tendinitis or bursitis or synovitis or sprain* or strain*).tw. |
| 69 | Spin* management.ti,ab. |
| 70 | Low back pain.ti,ab. |
| 71 | (arthriti* or osteoarthriti* or osteoporo* or bone loss).tw. |
| 72 | ankle/ or hip/ or knee/ |
| 73 | elbow/ or wrist/ or shoulder/ |
| 74 | elbow joint/ or exp hand joints/ or hip joint/ or knee joint/ or sacroiliac joint/ or shoulder joint/ |
| 75 | (spine or spinal).tw. |
| 76 | Tennis Elbow.mp. or Tennis Elbow/ |
| 77 | Golf Elbow.mp. |
| 78 | Medial epicondylitis.mp. or Elbow Tendinopathy/ |
| 79 | Lateral epicondylitis.mp. |
| 80 | 47 or 48 or 49 or 50 or 51 or 52 or 53 or 54 or 55 or 56 or 57 or 58 or 59 or 60 or 61 or 62 or 63 or 64 or 65 or 66 or 67 or 68 or 69 or 70 or 71 or 72 or 73 or 74 or 75 or 76 or 77 or 78 or 79 |
| 81 | randomized controlled trial.pt |
| 82 | controlled clinical trial.pt. |
| 83 | randomi?ed.ab. |
| 84 | placebo.ab. |
| 85 | drug therapy.fs. |
| 86 | randomly.ab. |
| 87 | trial.ab. |
| 88 | groups.ab. |
| 89 | or/81-88 |
| 90 | exp animals/ not humans.sh. |
| 91 | 89 not 90 |
| 92 | 8 and 46 and 80 and 91 |

**2. Embase: Embase 1996 to 2021 Week 03**

| 1 | exp Translational Research/ or Information Dissemination/ or Attitudes to health/ |
| --- | --- |
| 2 | exp Practice Guidelines/ or Health care planning/ or Knowledge Management/ or Continuing education/ |
| 3 | (KT adj1 (intervention or interventions or plan or plans or policy or policies or strategy or strategies or uptake)).mp. |
| 4 | ((knowledge or research or information or evidence or science or finding*) adj1 (translation or transfer or exchange or action or practice or decision or implementation or management or dissemination or application or share or sharing or uptake or utili?ation or mobile?ation or integration or communication or adoption or diffusion or brokering or creation)).mp. |
| 5 | (knowledge to action or research to practice or diffusion of innovations or scale up or translational research or translation of research findings or implementation or continuing education or organi*ational innovation or complex intervention or behavio*r change intervention* or "technology transfer").mp. |
| 6 | ((Continuing adj2 professional adj2 development) or (implementation adj2 science)).mp. |
| 7 | (intervention? adj2 (complex or education or multifacet* or multi-facet* or organi?ation* or tailor* or target* or interdisciplin* or multi-disciplin* or multidiscipline* or team* or evidence or evidence-based or evidence-driven)).mp. |
| 8 | 1 or 2 or 3 or 4 or 5 or 6 or 7 |
| 9 | Physiotherapy/ |
| 10 | Heart rehabilitation.mp. or heart rehabilitation/ |
| 11 | pulmonary rehabilitation.mp. or chronic obstructive lung disease/ or pulmonary rehabilitation/ |
| 12 | Neurological Rehabilitation.mp. or neurorehabilitation/ |
| 13 | osteopathic manipulation/ or chiropractic manipulation/ or musculoskeletal manipulation/ or spine manipulation/ or orthopedic manipulation/ |
| 14 | Mobilization.mp. |
| 15 | osteopathic medicine/ or Osteopathic.mp. or osteopathic manipulation/ or osteopathic physician/ |
| 16 | Exercise Therapy.mp. or kinesiotherapy/ |
| 17 | (physical therap* or physiotherap*).mp. |
| 18 | Occupational therapy/ or occupational therap*.mp. |
| 19 | Chiropract*.tw. |
| 20 | Chiroprax*.tw. |
| 21 | Chiropractic.mp. |
| 22 | OMT.mp. |
| 23 | Osteopath*.ti,ab. |
| 24 | (Osteopath* adj1 manipulat$).mp. |
| 25 | Manual therap*.mp. |
| 26 | Sport* therap*.mp. |
| 27 | Sport physician*.mp. |
| 28 | Sports medicine/ |
| 29 | Massage therap*.mp. |
| 30 | kinesiolog*.mp. |
| 31 | Physiatr*.mp. |
| 32 | Physical medicine.mp. |
| 33 | (physical adj2 medicine).mp. |
| 34 | Rehabilitation medicine.mp. |
| 35 | Orthoped*.mp. |
| 36 | Orthopaed*.mp. |
| 37 | Orthopod*.mp. |
| 38 | Doctor of Podiatric Medicine.mp. |
| 39 | Podiatr*.mp. |
| 40 | Chiropod*.mp. |
| 41 | Hand therap*.mp. |
| 42 | Foot therap*.mp. |
| 43 | 9 or 10 or 11 or 12 or 13 or 14 or 15 or 16 or 17 or 18 or 19 or 20 or 21 or 22 or 23 or 24 or 25 or 26 or 27 or 28 or 29 or 30 or 31 or 32 or 33 or 34 or 35 or 36 or 37 or 38 or 39 or 40 or 41 or 42 |
| 44 | musculoskeletal disease.mp. or musculoskeletal disease/ |
| 45 | Musculoskeletal adj2 (condition or abnorm$ or disorder or defect).ti,ab. |
| 46 | exp backache/ |
| 47 | exp Tennis Elbow/ |
| 48 | exp tendinitis/ |
| 49 | exp whiplash injury/ |
| 50 | exp intervertebral disk hernia/ or exp sciatica/ or exp lumbar disk hernia/ or exp low back pain/ |
| 51 | exp spine/ |
| 52 | exp Joint dislocation/ |
| 53 | exp Joint instability/ |
| 54 | exp spondylarthritis/ |
| 55 | exp osteoarthritis/ or exp hand osteoarthritis/ or exp experimental osteoarthritis/ or exp hip osteoarthritis/ or exp knee osteoarthritis/ |
| 56 | exp Osteoporosis/ |
| 57 | (pain adj1 (neck or back or shoulder? or elbow? or forearm? or wrist? or hand? or arm? or hip? or knee? or ankle? or leg? or foot or feet)).ti,ab. |
| 58 | (epicondylitis or tendonitis or tendinitis or bursitis or synovitis or sprain* or strain*).ti,ab. |
| 59 | Spin* management.ti,ab. |
| 60 | Low back pain.ti,ab. |
| 61 | (arthriti* or osteoarthriti* or osteoporo* or bone loss).ti,ab. |
| 62 | Golf Elbow.ti,ab. |
| 63 | exp epicondylitis/ or exp medial epicondylitis/ |
| 64 | Lateral epicondylitis.ti,ab. |
| 65 | 44 or 45 or 46 or 47 or 48 or 49 or 50 or 51 or 52 or 53 or 54 or 55 or 56 or 57 or 58 or 59 or 60 or 61 or 62 or 63 or 64 |
| 66 | Randomized controlled trial/ |
| 67 | Controlled clinical study/ |
| 68 | random$.ti,ab. |
| 69 | randomization/ |
| 70 | intermethod comparison/ |
| 71 | placebo.ti,ab. |
| 72 | (compare or compared or comparison).ti. |
| 73 | ((evaluated or evaluate or evaluating or assessed or assess) and (compare or compared or comparing or comparison)).ab. |
| 74 | (open adj label).ti,ab. |
| 75 | ((double or single or doubly or singly) adj (blind or blinded or blindly)).ti,ab. |
| 76 | double blind procedure/ |
| 77 | parallel group$1.ti,ab. |
| 78 | (crossover or cross over).ti,ab. |
| 79 | ((assign$ or match or matched or allocation) adj5 (alternate or group$1 or intervention$1 or patient$1 or subject$1 or participant$1)).ti,ab. |
| 80 | (assigned or allocated).ti,ab. |
| 81 | (controlled adj7 (study or design or trial)).ti,ab. |
| 82 | (volunteer or volunteers).ti,ab. |
| 83 | human experiment/ |
| 84 | trial.ti. |
| 85 | or/66-84 |
| 86 | (random$ adj sampl$ adj7 ("cross section$" or questionnaire$1 or survey$ or database$1)).ti,ab. not (comparative study/ or controlled study/ or randomi?ed controlled.ti,ab. or randomly assigned.ti,ab.) |
| 87 | Cross-sectional study/ not (randomized controlled trial/ or controlled clinical study/ or controlled study/ or randomi?ed controlled.ti,ab. or control group$1.ti,ab.) |
| 88 | (((case adj control$) and random$) not randomi?ed controlled).ti,ab. |
| 89 | (Systematic review not (trial or study)).ti. |
| 90 | (nonrandom$ not random$).ti,ab. |
| 91 | "Random field$".ti,ab. |
| 92 | (random cluster adj3 sampl$).ti,ab. |
| 93 | (review.ab. and review.pt.) not trial.ti. |
| 94 | "we searched".ab. and (review.ti. or review.pt.) |
| 95 | "update review".ab. |
| 96 | (databases adj4 searched).ab. |
| 97 | (rat or rats or mouse or mice or swine or porcine or murine or sheep or lambs or pigs or piglets or rabbit or rabbits or cat or cats or dog or dogs or cattle or bovine or monkey or monkeys or trout or marmoset$1).ti. and animal experiment/ |
| 98 | Animal experiment/ not (human experiment/ or human/) |
| 99 | or/86-98 |
| 100 | 85 not 99 |
| 101 | 8 and 43 and 65 and 100 |

**3. PsycINFO: APA PsycInfo 1987 to January Week 4 2021**

| 1 | Exp Interdisciplinary Research/ or Medical Education/ or Scientific Communication/ or Information Dissemination/ or Health Attitudes/ |
| --- | --- |
| 2 | Exp Evidence Based Practice/ or Treatment Guidelines/ or Knowledge Management/ or Continuing education/ |
| 3 | (KT adj1 (intervention or interventions or plan or plans or policy or policies or strategy or strategies or uptake)).mp. |
| 4 | ((knowledge or research or information or evidence or science or finding*) adj1 (translation or transfer or exchange or action or practice or decision or implementation or management or dissemination or application or share or sharing or uptake or utili?ation or mobile?ation or integration or communication or adoption or diffusion or brokering or creation)).mp. |
| 5 | (knowledge to action or research to practice or diffusion of innovations or scale up or translational research or translation of research findings or implementation or continuing education or organi*ational innovation or complex intervention or behavio*r change intervention* or "technology transfer").mp. |
| 6 | ((Continuing adj2 professional adj2 development) or (implementation adj2 science)).mp. |
| 7 | (intervention? adj2 (complex or education or multifacet* or multi-facet* or organi?ation* or tailor* or target* or interdisciplin* or multi-disciplin* or multidiscipline* or team* or evidence or evidence-based or evidence-driven)).mp. |
| 8 | 1 or 2 or 3 or 4 or 5 or 6 or 7 |
| 9 | exp Physical Therapy/ or Rehabilitation/ or Neurorehabilitation/ |
| 10 | Mobilization.mp. |
| 11 | exp Osteopathic Medicine/ |
| 12 | (physical therap* or physiotherap*).mp. |
| 13 | Occupational therapy/ or occupational therap*.mp. |
| 14 | Chiropract*.tw. |
| 15 | Chiroprax*.tw. |
| 16 | Chiropractic.mp. |
| 17 | OMT.mp. |
| 18 | Osteopath*.ti,ab. |
| 19 | (Osteopath* adj1 manipulat$).mp. |
| 20 | Manual therap*.mp. |
| 21 | Sport* therap*.mp. |
| 22 | Sport physician*.mp. |
| 23 | Sports medicine/ |
| 24 | Massage therap*.mp. |
| 25 | kinesiolog*.mp. |
| 26 | Physiatr*.mp. |
| 27 | Physical medicine.mp. |
| 28 | (physical adj2 medicine).mp. |
| 29 | Rehabilitation medicine.mp. |
| 30 | Orthoped*.mp. |
| 31 | Orthopaed*.mp. |
| 32 | Orthopod*.mp. |
| 33 | Doctor of Podiatric Medicine.mp. |
| 34 | Podiatr*.mp. |
| 35 | Chiropod*.mp. |
| 36 | Hand therap*.mp. |
| 37 | Foot therap*.mp. |
| 38 | 9 or 10 or 11 or 12 or 13 or 14 or 15 or 16 or 17 or 18 or 19 or 20 or 21 or 22 or 23 or 24 or 25 or 26 or 27 or 28 or 29 or 30 or 31 or 32 or 33 or 34 or 35 or 36 or 37 |
| 39 | exp Musculoskeletal Disorders/ or Musculoskeletal Disease.ti,ab. |
| 40 | Musculoskeletal adj2 (condition or abnorm$ or disorder or defect).ti,ab. |
| 41 | exp back pain/ |
| 42 | exp Whiplash/ |
| 43 | exp Joint Disorders/ |
| 44 | exp Arthritis/ |
| 45 | exp Osteoporosis/ |
| 46 | exp Spinal Column/ |
| 47 | exp Spinal Cord Injuries/ |
| 48 | (pain adj1 (neck or back or shoulder* or elbow* or forearm* or wrist* or hand* or arm* or hip* or knee* or ankle* or leg* or foot or feet)).ti,ab. |
| 49 | (epicondylitis or tendonitis or tendinitis or bursitis or synovitis or sprain* or strain*).ti,ab. |
| 50 | Spin* management.ti,ab. |
| 51 | Low back pain.ti,ab. |
| 52 | (arthriti* or osteoarthriti* or osteoporo* or bone loss).ti,ab. |
| 53 | Exp Joint Disorders/ |
| 54 | 39 or 40 or 41 or 42 or 43 or 44 or 45 or 46 or 47 or 48 or 49 or 50 or 51 or 52 or 53 |
| 55 | Treatment Effectiveness Evaluation/ or exp Treatment Outcomes/ |
| 56 | placebo/ |
| 57 | Followup Studies/ |
| 58 | (placebo* or random* or "comparative stud*" or (clinical adj3 trial*) or (research adj3 design) or (evaluat* adj3 stud*) or (prospectiv* adj3 stud*) or ((singl* or doubl* or trebl* or tripl*) adj3 (blind* or mask*))).tw,id. |
| 59 | 55 or 56 or 57 or 58 |
| 60 | 8 and 38 and 54 and 59 |

**4. CINAHL– Cumulative Index to Nursing & Allied Health Literature:**

| 1 | (MM "Attitude to Health") OR (MM "Practice Guidelines") OR (MM "Professional Practice, Research-Based") OR (MM "Professional Practice, TheORy-Based") OR (MM "Continuing Education Providers") OR (MM "Education, Medical, Continuing") OR (MM "Diffusion of Innovation") OR "Diffusion of Innovation" |
| --- | --- |
| 2 | TI ((KT N1 intervention*) OR (KT N1 plan*) OR (KT N1 policy) OR (KT N1 policies) OR (KT N1 strateg*)) |
| 3 | AB ((KT N1 intervention*) OR (KT N1 plan*) OR (KT N1 policy) OR (KT N1 policies) OR (KT N1 strateg*)) |
| 4 | TI ((knowledge N1 translation) OR (knowledge N1 transfer) OR (knowledge N1 exchange) OR (knowledge N1 action) OR (knowledge N1 practice) OR (knowledge N1 decision) OR (knowledge N1 implementation) OR (knowledge N1 management) OR (knowledge N1 dissemination) OR (knowledge N1 application) OR (knowledge N1 share) OR (knowledge N1 sharing) OR (knowledge N1 uptake) OR (knowledge N1 utili?ation) OR (knowledge N1 mobile?ation) OR (knowledge N1 integration) OR (knowledge N1 communication) OR (knowledge N1 adoption) OR (knowledge N1 diffusion) OR (knowledge N1 brokering) OR (knowledge N1 creation) OR (research N1 translation) OR (research N1 transfer) OR (research N1 exchange) OR (research N1action) OR (research N1 practice) OR (research N1 decision) OR (research N1 implementation) OR (research N1 management) OR (research N1 dissemination) OR (research N1 application) OR (research N1 share) OR (research N1 sharing) OR (research N1 uptake) OR (research N1 utili?ation) OR (research N1 mobile?ation) OR (research N1 integration) OR (research N1 communication) OR (research N1 adoption) OR (research N1 diffusion) OR (research N1 brokering) OR (research N1 creation) OR (infORmation N1 translation) OR (infORmation N1 transfer) OR (infORmation N1 exchange) OR (infORmation N1 action) OR (infORmation N1 practice) OR (infORmation N1 decision) OR (infORmation N1 implementation) OR (infORmation N1 management) OR (infORmation N1 dissemination) OR (infORmation N1 application) OR (infORmation N1 share) OR (infORmation N1 sharing) OR (infORmation N1 uptake) OR (infORmation N1 utili?ation) OR (infORmation N1 mobile?ation) OR (infORmation N1 integration) OR (infORmation N1 communication) OR (infORmation N1 adoption) OR (infORmation N1 diffusion) OR (infORmation N1 brokering) OR (infORmation N1 creation) OR (evidence N1 translation) OR (evidence N1 transfer) OR (evidence N1 exchange) OR (evidence N1 action) OR (evidence N1 practice) OR (evidence N1 decision) OR (evidence N1 implementation) OR (evidence N1 management) OR (evidence N1 dissemination) OR (evidence N1 application) OR (evidence N1 share) OR (evidence N1 sharing) OR (evidence N1 uptake) OR (evidence N1 utili?ation) OR (evidence N1 mobile?ation) OR (evidence N1 integration) OR (evidence N1 communication) OR (evidence N1 adoption) OR (evidence N1 diffusion) OR (evidence N1 brokering) OR (evidence N1 creation) OR (science N1 translation) OR (science N1 transfer) OR (science N1 exchange) OR (science N1 action) OR (science N1 practice) OR (science N1 decision) OR (science N1 implementation) OR (science N1 management) OR (science N1 dissemination) OR (science N1 application) OR (science N1 share) OR (science N1 sharing) OR (science N1 uptake) OR (science N1 utili?ation) OR (science N1 mobile?ation) OR (science N1 integration) OR (science N1 communication) OR (science N1 adoption) OR (science N1 diffusion) OR (science N1 brokering) OR (science N1 creation) OR (finding* N1 translation) OR (finding* N1 transfer) OR (finding* N1 exchange) OR (finding* N1 action) OR (finding* N1 practice) OR (finding* N1 decision) OR (finding* N1 implementation) OR (finding* N1 management) OR (finding* N1 dissemination) OR (finding* N1 application) OR (finding* N1 share) OR (finding* N1 sharing) OR (finding* N1 uptake) OR (finding* N1 utili?ation) OR (finding* N1 mobile?ation) OR (finding* N1 integration) OR (finding* N1 communication) OR (finding* N1 adoption) OR (finding* N1 diffusion) OR (finding* N1 brokering) OR (finding* N1 creation)) |
| 5 | AB ((knowledge N1 translation) OR (knowledge N1 transfer) OR (knowledge N1 exchange) OR (knowledge N1 action) OR (knowledge N1 practice) OR (knowledge N1 decision) OR (knowledge N1 implementation) OR (knowledge N1 management) OR (knowledge N1 dissemination) OR (knowledge N1 application) OR (knowledge N1 share) OR (knowledge N1 sharing) OR (knowledge N1 uptake) OR (knowledge N1 utili?ation) OR (knowledge N1 mobile?ation) OR (knowledge N1 integration) OR (knowledge N1 communication) OR (knowledge N1 adoption) OR (knowledge N1 diffusion) OR (knowledge N1 brokering) OR (knowledge N1 creation) OR (research N1 translation) OR (research N1 transfer) OR (research N1 exchange) OR (research N1action) OR (research N1 practice) OR (research N1 decision) OR (research N1 implementation) OR (research N1 management) OR (research N1 dissemination) OR (research N1 application) OR (research N1 share) OR (research N1 sharing) OR (research N1 uptake) OR (research N1 utili?ation) OR (research N1 mobile?ation) OR (research N1 integration) OR (research N1 communication) OR (research N1 adoption) OR (research N1 diffusion) OR (research N1 brokering) OR (research N1 creation) OR (infORmation N1 translation) OR (infORmation N1 transfer) OR (infORmation N1 exchange) OR (infORmation N1 action) OR (infORmation N1 practice) OR (infORmation N1 decision) OR (infORmation N1 implementation) OR (infORmation N1 management) OR (infORmation N1 dissemination) OR (infORmation N1 application) OR (infORmation N1 share) OR (infORmation N1 sharing) OR (infORmation N1 uptake) OR (infORmation N1 utili?ation) OR (infORmation N1 mobile?ation) OR (infORmation N1 integration) OR (infORmation N1 communication) OR (infORmation N1 adoption) OR (infORmation N1 diffusion) OR (infORmation N1 brokering) OR (infORmation N1 creation) OR (evidence N1 translation) OR (evidence N1 transfer) OR (evidence N1 exchange) OR (evidence N1 action) OR (evidence N1 practice) OR (evidence N1 decision) OR (evidence N1 implementation) OR (evidence N1 management) OR (evidence N1 dissemination) OR (evidence N1 application) OR (evidence N1 share) OR (evidence N1 sharing) OR (evidence N1 uptake) OR (evidence N1 utili?ation) OR (evidence N1 mobile?ation) OR (evidence N1 integration) OR (evidence N1 communication) OR (evidence N1 adoption) OR (evidence N1 diffusion) OR (evidence N1 brokering) OR (evidence N1 creation) OR (science N1 translation) OR (science N1 transfer) OR (science N1 exchange) OR (science N1 action) OR (science N1 practice) OR (science N1 decision) OR (science N1 implementation) OR (science N1 management) OR (science N1 dissemination) OR (science N1 application) OR (science N1 share) OR (science N1 sharing) OR (science N1 uptake) OR (science N1 utili?ation) OR (science N1 mobile?ation) OR (science N1 integration) OR (science N1 communication) OR (science N1 adoption) OR (science N1 diffusion) OR (science N1 brokering) OR (science N1 creation) OR (finding* N1 translation) OR (finding* N1 transfer) OR (finding* N1 exchange) OR (finding* N1 action) OR (finding* N1 practice) OR (finding* N1 decision) OR (finding* N1 implementation) OR (finding* N1 management) OR (finding* N1 dissemination) OR (finding* N1 application) OR (finding* N1 share) OR (finding* N1 sharing) OR (finding* N1 uptake) OR (finding* N1 utili?ation) OR (finding* N1 mobile?ation) OR (finding* N1 integration) OR (finding* N1 communication) OR (finding* N1 adoption) OR (finding* N1 diffusion) OR (finding* N1 brokering) OR (finding* N1 creation)) |
| 6 | TI ((knowledge to action) OR (implementation) OR (research to practice) OR (diffusion of innovations) OR (scale up) OR (translational research) OR (translation of research findings) OR (continuing education) OR (ORgani*ational innovation) OR (complex intervention) OR (behavio*r change intervention*) OR (technology transfer)) OR AB ((knowledge to action) OR (implementation) OR (research to practice) OR (diffusion of innovations) OR (scale up) OR (translational research) OR (translation of research findings) OR (continuing education) OR (ORgani*ational innovation) OR (complex intervention) OR (behavio*r change intervention*) OR (technology transfer)) |
| 7 | TI ((Continuing N2 professional N2 development) OR (implementation N2 science)) OR AB ((Continuing N2 professional N2 development) OR (implementation N2 science)) |
| 8 | TI ((intervention? N2 complex) OR (intervention? N2 education*) OR (intervention? N2 multifacet*) OR (intervention? N2 multi-facet*) OR (intervention? N2 ORgani?ation*) OR (intervention? N2 tailOR*) OR (intervention? N2 target*) OR (intervention? N2 interdisciplin*) OR (intervention? N2 multi-disciplin*) OR (intervention? N2 multidiscipline*) OR (intervention? N2 evidence-based) OR (intervention? N2 evidence-driven)) OR AB ((intervention? N2 complex) OR (intervention? N2 education*) OR (intervention? N2 multifacet*) OR (intervention? N2 multi-facet*) OR (intervention? N2 ORgani?ation*) OR (intervention? N2 tailOR*) OR (intervention? N2 target*) OR (intervention? N2 interdisciplin*) OR (intervention? N2 multi-disciplin*) OR (intervention? N2 multidiscipline*) OR (intervention? N2 evidence-based) OR (intervention? N2 evidence-driven)) |
| 9 | 1 or 2 or 3 or 4 or 5 or 6 or 7 or 8 |
| 10 | (MH "Rehabilitation+") OR (MH "Occupational Therapy+") OR (MH "Occupational Therap*") OR (MH "Physical Therapy+") OR (MH "Physical Therap*") OR (MH "Manipulation, Chiropractic") OR (MH "Manual Therapy") OR (MH "Manipulation, ORthopedic") OR (MH "Chiropract*") OR (MH "Osteopathic Medicine") OR (MH "Manipulation, Osteopathic") OR (MH "Osteopaths") OR (MH "Physicians, SpORts Team") OR (MH "SpORts Medicine") OR (MH "Exercise Physiolog*") OR (MH "Therapeutic Exercise*") OR (MH "Massage Therap*") OR (MH "Kinesiolog*") |
| 11 | (MH "Physical Medicine") OR (MH "Physical Therapist Attitudes") OR (MH "Occupational Therapist Attitudes") OR (MH "Occupational Medicine") OR (MH "Hand Therapy") OR (MH "Foot Therapy") OR (MH "Podiatry Practice") OR (MH "Podiatr*") OR (MH "Rehabilitation, Cardiac+") OR (MH "Rehabilitation, Pulmonary+") OR (MH "Rehabilitation, Pediatric") OR (MH “neurological rehabilitation") |
| 12 | 10 or 11 |
| 13 | (MM "Joint Mobilization") OR "mobilization" |
| 14 | (MM "Manipulation, ORthopedic") OR (MM "Manipulation, Chiropractic") OR (MM "Manipulation, Osteopathic") OR "Manipulation" |
| 15 | "(physical N3 medicine)" OR (MM "Physical Medicine") |
| 16 | (MH "Low Back Pain") OR (MH "Back Pain") OR (MH "Neck Pain") |
| 17 | (MH "Shoulder Pain") |
| 18 | (MM "Golf Elbow") OR (MM "Elbow Fractures") OR (MM "Elbow Dislocation") OR (MM "Elbow Injuries") OR (MM "Medial Epicondyle Apophysitis") OR (MM "Cubital Tunnel Syndrome") OR (MM "Tennis Elbow") OR (MM "Elbow Pain") OR (MM "lateral epicondylitis") |
| 19 | (MH "Whiplash Injuries") |
| 20 | (MH "Sciatica") |
| 21 | (MH "Intervertebral Disk Displacement") OR (MH "Intervertebral Disk") |
| 22 | (MH "epicondylitis") OR (MH "Tendinopathy") OR (MH "Bursitis") OR (MH "Synovitis") OR (MH "Sprains and Strains") OR (MH "Joint Diseases") OR (MH "Spinal Diseases") OR (MH "Spondylarthritis") OR (MH "Osteoarthritis") OR (MH "OsteopORosis") OR (MH "Arthritis") OR (MH "osteoarthritis") OR ("osteopORo*") |
| 23 | "bone loss*" OR (MH "Bone Density") |
| 24 | (MH "Ankle") OR (MH "hip") OR (MH "knee") OR (MH "elbow") OR (MH "wrist") OR (MH "shoulder") OR (MH "hip Joint") OR (MH "Elbow Joint") OR (MH "hand Joint") OR (MH "knee Joint") OR (MH "sacroiliac Joint") OR (MH "shoulder Joint") |
| 25 | (MH "Cervical Vertebrae") OR (MH "Lumbar Vertebrae") OR (MH "ThORacic Vertebrae") OR (MH "Spondylosis") |
| 26 | (MH "Intervertebral Disk") OR (MH "Back") OR (MH "Spine") |
| 27 | (MH "Musculoskeletal Diseases") OR (MH "Joint dislocation") OR (MH "Joint instability") OR (MH "Spine management") |
| 28 | TI ((Musculoskeletal N2 condition) OR (Musculoskeletal N2 abnORm$) OR (Musculoskeletal N2 disORder) OR (Musculoskeletal N2 defect)) |
| 29 | AB ((Musculoskeletal N2 condition) OR (Musculoskeletal N2 abnORm$) OR (Musculoskeletal N2 disORder) OR (Musculoskeletal N2 defect)) |
| 30 | TI ((pain N2 neck) OR (pain N2 back) OR (pain N2 shoulder?) OR (pain N2 elbow?) OR (pain N2 fORearm?) OR (pain N2 wrist?) OR (pain N2 hand?) OR (pain N2 arm?) OR (pain N2 hip?) OR (pain N2 knee?) OR (pain N2 ankle?) OR (pain N2 leg?) OR (pain N2 foot) OR (pain N2 feet)) OR AB ((pain N2 neck) OR (pain N2 back) OR (pain N2 shoulder?) OR (pain N2 elbow?) OR (pain N2 fORearm?) OR (pain N2 wrist?) OR (pain N2 hand?) OR (pain N2 arm?) OR (pain N2 hip?) OR (pain N2 knee?) OR (pain N2 ankle?) OR (pain N2 leg?) OR (pain N2 foot) OR (pain N2 feet)) |
| 31 | 13 or 14 or 15 or 16 or 17 or 18 or 19 or 20 or 21 or 22 or 23 or 24 or 25 or 26 or 27 or 28 or 29 or 30 |
| 32 | (MH randomized controlled trials OR MH double‐blind studies OR MH single‐blind studies OR MH random assignment OR MH pretest‐posttest design OR MH cluster sample OR TI (randomised OR randomized) OR AB (random*) OR TI (trial) OR (MH (sample size) AND AB (assigned OR allocated OR control)) OR MH (placebos) OR PT (randomized controlled trial) OR AB (control W5 group) OR MH (crossover design) OR MH (comparative studies) OR AB (cluster W3 RCT) NOT ((MH animals+ OR MH (animal studies) OR TI (animal model*)) NOT MH (human)) |
| 33 | 9 and 12 and 31 and 32 |

**5. Cochrane Central**

| 1 | (KT NEAR/1 intervention*):ti,ab or (KT NEAR/1 plan*):ti,ab or (KT NEAR/1 policy):ti,ab or (KT NEAR/1 policies):ti,ab or (KT NEAR/1 strateg*):ti,ab |
| --- | --- |
| 2 | (knowledge NEAR/1 translation):ti,ab or (knowledge NEAR/1 transfer):ti,ab or (knowledge NEAR/1 exchange):ti,ab or (knowledge NEAR/1 action):ti,ab or (knowledge NEAR/1 practice):ti,ab or (knowledge NEAR/1 decision):ti,ab or (knowledge NEAR/1 implementation):ti,ab or (knowledge NEAR/1 management):ti,ab or (knowledge NEAR/1 dissemination):ti,ab or (knowledge NEAR/1 application):ti,ab or (knowledge NEAR/1 share):ti,ab or (knowledge NEAR/1 sharing):ti,ab or (knowledge NEAR/1 uptake):ti,ab or (knowledge NEAR/1 utili?ation):ti,ab or (knowledge NEAR/1 mobile?ation):ti,ab or (knowledge NEAR/1 integration):ti,ab or (knowledge NEAR/1 communication):ti,ab or (knowledge NEAR/1 adoption):ti,ab or (knowledge NEAR/1 diffusion):ti,ab or (knowledge NEAR/1 brokering):ti,ab or (knowledge NEAR/1 creation):ti,ab or (research NEAR/1 translation):ti,ab or (research NEAR/1 transfer):ti,ab or (research NEAR/1 exchange):ti,ab or (research NEAR/1action):ti,ab or (research NEAR/1 practice):ti,ab or (research NEAR/1 decision):ti,ab or (research NEAR/1 implementation):ti,ab or (research NEAR/1 management):ti,ab or (research NEAR/1 dissemination):ti,ab or (research NEAR/1 application):ti,ab or (research NEAR/1 share):ti,ab or (research NEAR/1 sharing):ti,ab or (research NEAR/1 uptake):ti,ab or (research NEAR/1 utili?ation):ti,ab or (research NEAR/1 mobile?ation):ti,ab or (research NEAR/1 integration):ti,ab or (research NEAR/1 communication):ti,ab or (research NEAR/1 adoption):ti,ab or (research NEAR/1 diffusion):ti,ab or (research NEAR/1 brokering):ti,ab or (research NEAR/1 creation):ti,ab or (information NEAR/1 translation):ti,ab or (information NEAR/1 transfer):ti,ab or (information NEAR/1 exchange):ti,ab or (information NEAR/1 action):ti,ab or (information NEAR/1 practice):ti,ab or (information NEAR/1 decision):ti,ab or (information NEAR/1 implementation):ti,ab or (information NEAR/1 management):ti,ab or (information NEAR/1 dissemination):ti,ab or (information NEAR/1 application):ti,ab or (information NEAR/1 share):ti,ab or (information NEAR/1 sharing):ti,ab or (information NEAR/1 uptake):ti,ab or (information NEAR/1 utili?ation):ti,ab or (information NEAR/1 mobile?ation):ti,ab or (information NEAR/1 integration):ti,ab or (information NEAR/1 communication):ti,ab or (information NEAR/1 adoption):ti,ab or (information NEAR/1 diffusion):ti,ab or (information NEAR/1 brokering):ti,ab or (information NEAR/1 creation):ti,ab or (evidence NEAR/1 translation):ti,ab or (evidence NEAR/1 transfer):ti,ab or (evidence NEAR/1 exchange):ti,ab or (evidence NEAR/1 action):ti,ab or (evidence NEAR/1 practice):ti,ab or (evidence NEAR/1 decision):ti,ab or (evidence NEAR/1 implementation):ti,ab or (evidence NEAR/1 management):ti,ab or (evidence NEAR/1 dissemination):ti,ab or (evidence NEAR/1 application):ti,ab or (evidence NEAR/1 share):ti,ab or (evidence NEAR/1 sharing):ti,ab or (evidence NEAR/1 uptake):ti,ab or (evidence NEAR/1 utili?ation):ti,ab or (evidence NEAR/1 mobile?ation):ti,ab or (evidence NEAR/1 integration):ti,ab or (evidence NEAR/1 communication):ti,ab or (evidence NEAR/1 adoption):ti,ab or (evidence NEAR/1 diffusion):ti,ab or (evidence NEAR/1 brokering):ti,ab or (evidence NEAR/1 creation):ti,ab or (science NEAR/1 translation):ti,ab or (science NEAR/1 transfer):ti,ab or (science NEAR/1 exchange):ti,ab or (science NEAR/1 action):ti,ab or (science NEAR/1 practice):ti,ab or (science NEAR/1 decision):ti,ab or (science NEAR/1 implementation):ti,ab or (science NEAR/1 management):ti,ab or (science NEAR/1 dissemination):ti,ab or (science NEAR/1 application):ti,ab or (science NEAR/1 share):ti,ab or (science NEAR/1 sharing):ti,ab or (science NEAR/1 uptake):ti,ab or (science NEAR/1 utili?ation):ti,ab or (science NEAR/1 mobile?ation):ti,ab or (science NEAR/1 integration):ti,ab or (science NEAR/1 communication):ti,ab or (science NEAR/1 adoption):ti,ab or (science NEAR/1 diffusion):ti,ab or (science NEAR/1 brokering):ti,ab or (science NEAR/1 creation):ti,ab or (finding* NEAR/1 translation):ti,ab or (finding* NEAR/1 transfer):ti,ab or (finding* NEAR/1 exchange):ti,ab or (finding* NEAR/1 action):ti,ab or (finding* NEAR/1 practice):ti,ab or (finding* NEAR/1 decision):ti,ab or (finding* NEAR/1 implementation):ti,ab or (finding* NEAR/1 management):ti,ab or (finding* NEAR/1 dissemination):ti,ab or (finding* NEAR/1 application):ti,ab or (finding* NEAR/1 share):ti,ab or (finding* NEAR/1 sharing):ti,ab or (finding* NEAR/1 uptake):ti,ab or (finding* NEAR/1 utili?ation):ti,ab or (finding* NEAR/1 mobile?ation):ti,ab or (finding* NEAR/1 integration):ti,ab or (finding* NEAR/1 communication):ti,ab or (finding* NEAR/1 adoption):ti,ab or (finding* NEAR/1 diffusion):ti,ab or (finding* NEAR/1 brokering):ti,ab or (finding* NEAR/1 creation):ti,ab |
| 3 | (knowledge to action):ti,ab or (implementation):ti,ab or (research to practice):ti,ab or (diffusion of innovations):ti,ab or (scale up):ti,ab or (translational research):ti,ab or (translation of research findings):ti,ab or (continuing education):ti,ab or (organi*ational innovation):ti,ab or (complex intervention):ti,ab or (behavio*r change intervention*):ti,ab or (technology transfer):ti,ab |
| 4 | (Continuing NEAR/2 professional NEAR/2 development):ti,ab or (implementation NEAR/2 science):ti,ab |
| 5 | (intervention? NEAR/1 complex):ti,ab or (intervention? NEAR/1 education*):ti,ab or (intervention? NEAR/1 multifacet*):ti,ab or (intervention? NEAR/1 multi-facet*):ti,ab or (intervention? NEAR/1 organi?ation*):ti,ab or (intervention? NEAR/1 tailor*):ti,ab or (intervention? NEAR/1 target*):ti,ab or (intervention? NEAR/1 interdisciplin*):ti,ab or (intervention? NEAR/1 multi-disciplin*):ti,ab or (intervention? NEAR/1 multidiscipline*):ti,ab or (intervention? NEAR/1 evidence-based):ti,ab or (intervention? NEAR/1 evidence-driven):ti,ab |
| 6 | 1 or 2 or 3 or 4 or 5 |
| 7 | Physical Therapy Modalities |
| 8 | "Physical Therapy (Specialty)" |
| 9 | "physical therapy procedure" |
| 10 | physical therap* or physiotherap* |
| 11 | occupational therap* |
| 12 | Manipulation, Chiropractic |
| 13 | chiropract*:ti,ab |
| 14 | Osteopathic Medicine:ti,ab |
| 15 | Manipulation, Osteopathic |
| 16 | Mobilization:ti,ab |
| 17 | OMT:ti,ab |
| 18 | Osteopathic Physician*:ti,ab |
| 19 | Osteopathic Medicine*:ti,ab |
| 20 | Osteopath*:ti,ab |
| 21 | manual therap*:ti,ab |
| 22 | athletic therap*:ti,ab |
| 23 | sport* therap*:ti,ab |
| 24 | sport physician*:ti,ab |
| 25 | (Sport* near/2 (medicine or therap*)):ti,ab |
| 26 | Exercise physiolog*:ti,ab |
| 27 | Exercise Therap*:ti,ab |
| 28 | kinesiolog*:ti,ab |
| 29 | Physiatr*:ti,ab |
| 30 | Physical and Rehabilitation Medicine:ti,ab |
| 31 | Physical medicine:ti,ab |
| 32 | (physical near/3 medicine):ti,ab |
| 33 | rehabilitation medicine:ti,ab |
| 34 | orthoped*:ti,ab |
| 35 | orthopaed*:ti,ab |
| 36 | Podiatr*:ti,ab |
| 37 | hand therap*:ti,ab |
| 38 | foot therap*:ti,ab |
| 39 | Chiropod*:ti,ab |
| 40 | "cardiac rehabilitation" |
| 41 | "pediatric rehabilitation" |
| 42 | "neurological rehabilitation" |
| 43 | "pulmonary rehabilitation" |
| 44 | 7 or 8 or 9 or 10 or 11 or 12 or 13 or 14 or 15 or 16 or 17 or 18 or 19 or 20 or 21 or 22 or 23 or 24 or 25 or 26 or 27 or 28 or 29 or 30 or 31 or 32 or 33 or 34 or 35 or 36 or 37 or 38 or 39 or 40 or 41 or 42 or 43 |
| 45 | Low back pain:ti,ab |
| 46 | Tennis Elbow:ti,ab |
| 47 | Golf Elbow:ti,ab |
| 48 | Medial epicondylitis:ti,ab |
| 49 | Lateral epicondylitis:ti,ab |
| 50 | Tendinopathy:ti,ab |
| 51 | Whiplash Injuries:ti,ab |
| 52 | Sciatica:ti,ab |
| 53 | Intervertebral Disk Displacement:ti,ab |
| 54 | (pain near/2 (neck or back or shoulder* or elbow* or forearm* or wrist* or hand* or arm* or hip* or knee* or ankle* or leg* or foot or feet)):ti,ab |
| 55 | (epicondylitis or tendonitis or tendinitis or bursitis or synovitis or sprain* or strain*):ti,ab |
| 56 | Joint Diseases:ti,ab |
| 57 | Spinal Diseases:ti,ab |
| 58 | Spondylarthritis:ti,ab |
| 59 | osteoarthritis:ti,ab |
| 60 | Osteoporosis:ti,ab |
| 61 | (arthriti* or osteoarthriti* or osteoporo* or bone loss*):ti,ab |
| 62 | Musculoskeletal disorder*:ti,ab |
| 63 | (Musculoskeletal near/2 condition):ti,ab or (Musculoskeletal near/2 abnorm$):ti,ab or (Musculoskeletal near/2 disorder):ti,ab or (Musculoskeletal near/2 defect):ti,ab |
| 64 | 45 or 46 or 47 or 48 or 49 or 50 or 51 or 52 or 53 or 54 or 55 or 56 or 57 or 58 or 59 or 60 or 61 or 62 or 63 |
| 65 | 6 and 44 and 64 = 2914 |

**Appendix (2): Expert Recommendations for Implementing Change (ERIC) classification**

| Source | Concept | Definition or Description |
| --- | --- | --- |
| Use evaluative and iterative strategies | Assess for readiness and identify barriers and facilitators | Assess various aspects of an organization to determine its degree of readiness to implement, barriers that may impede implementation, and strengths that can be used in the implementation effort |
|  | Audit and provide feedback | Collect and summarize clinical performance data over a specified time period and give it to clinicians and administrators to monitor, evaluate, and modify provider behavior |
|  | Conduct cyclical small tests of change | Implement changes in a cyclical fashion using small tests of change before taking changes system-wide. Tests of change benefit from systematic measurement, and results of the tests of change are studied for insights on how to do better. This process continues serially over time, and refinement is added with each cycle |
|  | Conduct local needs assessment | Collect and analyze data related to the need for the innovation |
|  | Develop a formal implementation blueprint | Develop a formal implementation blueprint that includes all goals and strategies. The blueprint should include the following: 1) aim/purpose of the implementation; 2) scope of the change (*e.g.*, what organizational units are affected); 3) timeframe and milestones; and 4) appropriate performance/progress measures. Use and update this plan to guide the implementation effort over time |
|  | Develop and implement tools for quality monitoring | Develop, test, and introduce into quality-monitoring systems the right input—the appropriate language, protocols, algorithms, standards, and measures (of processes, patient/consumer outcomes, and implementation outcomes) that are often specific to the innovation being implemented |
|  | Develop and organize quality monitoring systems | Develop and organize systems and procedures that monitor clinical processes and/or outcomes for the purpose of quality assurance and improvement |
|  | Obtain and use patients/consumers and family feedback | Develop strategies to increase patient/consumer and family feedback on the implementation effort |
|  | Purposely reexamine the implementation | Monitor progress and adjust clinical practices and implementation strategies to continuously improve the quality of care |
|  | Stage implementation scale up | Phase implementation efforts by starting with small pilots or demonstration projects and gradually move to a system wide rollout |
| Provide interactive assistance | Centralize technical assistance | Develop and use a centralized system to deliver technical assistance focused on implementation issues |
|  | Facilitation | A process of interactive problem solving and support that occurs in a context of a recognized need for improvement and a supportive interpersonal relationship |
|  | Provide clinical supervision | Provide clinicians with ongoing supervision focusing on the innovation. Provide training for clinical supervisors who will supervise clinicians who provide the innovation |
|  | Provide local technical assistance | Develop and use a system to deliver technical assistance focused on implementation issues using local personnel |
| Adapt and tailor to context | Promote adaptability | Identify the ways a clinical innovation can be tailored to meet local needs and clarify which elements of the innovation must be maintained to preserve fidelity |
|  | Tailor strategies | Tailor the implementation strategies to address barriers and leverage facilitators that were identified through earlier data collection |
|  | Use data experts | Involve, hire, and/or consult experts to inform management on the use of data generated by implementation efforts |
|  | Use data warehousing techniques | Integrate clinical records across facilities and organizations to facilitate implementation across systems |
| Develop stakeholder interrelationships | Build a coalition | Recruit and cultivate relationships with partners in the implementation effort |
|  | Capture and share local knowledge | Capture local knowledge from implementation sites on how implementers and clinicians made something work in their setting and then share it with other sites |
|  | Conduct local consensus discussions | Include local providers and other stakeholders in discussions that address whether the chosen problem is important and whether the clinical innovation to address it is appropriate |
|  | Develop academic partnerships | Partner with a university or academic unit for the purposes of shared training and bringing research skills to an implementation project |
|  | Develop an implementation glossary | Develop and distribute a list of terms describing the innovation, implementation, and stakeholders in the organizational change |
|  | Identify and prepare champions | Identify and prepare individuals who dedicate themselves to supporting, marketing, and driving through an implementation, overcoming indifference or resistance that the intervention may provoke in an organization |
|  | Identify early adopters | Identify early adopters at the local site to learn from their experiences with the practice innovation |
|  | Inform local opinion leaders | Inform providers identified by colleagues as opinion leaders or “educationally influential” about the clinical innovation in the hopes that they will influence colleagues to adopt it |
|  | Involve executive boards | Involve existing governing structures (*e.g.*, boards of directors, medical staff boards of governance) in the implementation effort, including the review of data on implementation processes |
|  | Model and simulate change | Model or simulate the change that will be implemented prior to implementation |
|  | Obtain formal commitments | Obtain written commitments from key partners that state what they will do to implement the innovation |
|  | Organize clinician implementation team meetings | Develop and support teams of clinicians who are implementing the innovation and give them protected time to reflect on the implementation effort, share lessons learned, and support one another’s learning |
|  | Promote network weaving | Identify and build on existing high-quality working relationships and networks within and outside the organization, organizational units, teams, etc. to promote information sharing, collaborative problem-solving, and a shared vision/goal related to implementing the innovation |
|  | Recruit, designate, and train for leadership | Recruit, designate, and train leaders for the change effort |
|  | Use advisory boards and workgroups | Create and engage a formal group of multiple kinds of stakeholders to provide input and advice on implementation efforts and to elicit recommendations for improvements |
|  | Use an implementation advisor | Seek guidance from experts in implementation |
|  | Visit other sites | Visit sites where a similar implementation effort has been considered successful |
| Train and educate stakeholders | Conduct educational meetings | Hold meetings targeted toward different stakeholder groups (*e.g.*, providers, administrators, other organizational stakeholders, and community, patient/consumer, and family stakeholders) to teach them about the clinical innovation |
|  | Conduct educational outreach visits | Have a trained person meet with providers in their practice settings to educate providers about the clinical innovation with the intent of changing the provider’s practice |
|  | Conduct ongoing training | Plan for and conduct training in the clinical innovation in an ongoing way |
|  | Create a learning collaborative | Facilitate the formation of groups of providers or provider organizations and foster a collaborative learning environment to improve implementation of the clinical innovation |
|  | Develop educational materials | Develop and format manuals, toolkits, and other supporting materials in ways that make it easier for stakeholders to learn about the innovation and for clinicians to learn how to deliver the clinical innovation |
|  | Distribute educational materials | Distribute educational materials (including guidelines, manuals, and toolkits) in person, by mail, and/or electronically |
|  | Make training dynamic | Vary the information delivery methods to cater to different learning styles and work contexts, and shape the training in the innovation to be interactive |
|  | Provide ongoing consultation | Provide ongoing consultation with one or more experts in the strategies used to support implementing the innovation |
|  | Shadow other experts | Provide ways for key individuals to directly observe experienced people engage with or use the targeted practice change/innovation |
|  | Use train-the-trainer strategies | Train designated clinicians or organizations to train others in the clinical innovation |
|  | Work with educational institutions | Encourage educational institutions to train clinicians in the innovation |
| Support clinicians | Create new clinical teams | Change who serves on the clinical team, adding different disciplines and different skills to make it more likely that the clinical innovation is delivered (or is more successfully delivered) |
|  | Develop resource sharing agreements | Develop partnerships with organizations that have resources needed to implement the innovation |
|  | Facilitate relay of clinical data to providers | Provide as close to real-time data as possible about key measures of process/outcomes using integrated modes/channels of communication in a way that promotes use of the targeted innovation |
|  | Remind clinicians | Develop reminder systems designed to help clinicians to recall information and/or prompt them to use the clinical innovation |
|  | Revise professional roles | Shift and revise roles among professionals who provide care, and redesign job characteristics |
| Engage consumers | Increase demand | Attempt to influence the market for the clinical innovation to increase competition intensity and to increase the maturity of the market for the clinical innovation |
|  | Intervene with patients/consumers to enhance uptake and adherence | Develop strategies with patients to encourage and problem solve around adherence |
|  | Involve patients/consumers and family members | Engage or include patients/consumers and families in the implementation effort |
|  | Prepare patients/consumers to be active participants | Prepare patients/consumers to be active in their care, to ask questions, and specifically to inquire about care guidelines, the evidence behind clinical decisions, or about available evidence-supported treatments |
|  | Use mass media | Use media to reach large numbers of people to spread the word about the clinical innovation |
| Use financial strategies | Access new funding | Access new or existing money to facilitate the implementation |
|  | Alter incentive/allowance structures | Work to incentivize the adoption and implementation of the clinical innovation |
|  | Alter patient/consumer fees | Create fee structures where patients/consumers pay less for preferred treatments (the clinical innovation) and more for less-preferred treatments |
|  | Develop disincentives | Provide financial disincentives for failure to implement or use the clinical innovations |
|  | Fund and contract for the clinical innovation | Governments and other payers of services issue requests for proposals to deliver the innovation, use contracting processes to motivate providers to deliver the clinical innovation, and develop new funding formulas that make it more likely that providers will deliver the innovation |
|  | Make billing easier | Make it easier to bill for the clinical innovation |
|  | Place innovation on fee for service lists/formularies | Work to place the clinical innovation on lists of actions for which providers can be reimbursed (*e.g.*, a drug is placed on a formulary, a procedure is now reimbursable) |
|  | Use capitated payments | Pay providers or care systems a set amount per patient/consumer for delivering clinical care |
|  | Use other payment schemes | Introduce payment approaches (in a catch-all category) |
| Change infrastructure | Change accreditation or membership requirements | Strive to alter accreditation standards so that they require or encourage use of the clinical innovation. Work to alter membership organization requirements so that those who want to affiliate with the organization are encouraged or required to use the clinical innovation |
|  | Change liability laws | Participate in liability reform efforts that make clinicians more willing to deliver the clinical innovation |
|  | Change physical structure and equipment | Evaluate current configurations and adapt, as needed, the physical structure and/or equipment (*e.g.*, changing the layout of a room, adding equipment) to best accommodate the targeted innovation |
|  | Change record systems | Change records systems to allow better assessment of implementation or clinical outcomes |
|  | Change service sites | Change the location of clinical service sites to increase access |
|  | Create or change credentialing and/or licensure standards | Create an organization that certifies clinicians in the innovation or encourage an existing organization to do so. Change governmental professional certification or licensure requirements to include delivering the innovation. Work to alter continuing education requirements to shape professional practice toward the innovation |
|  | Mandate change | Have leadership declare the priority of the innovation and their determination to have it implemented |
|  | Start a dissemination organization | Identify or start a separate organization that is responsible for disseminating the clinical innovation. It could be a for-profit or non-profit organization |
|  | **Acceptability**; Satisfaction with various aspects of the innovation (e.g. content, complexity, comfort, delivery, and credibility) | Acceptability is the perception among implementation stakeholders that a given treatment, service, practice, or innovation is agreeable, palatable, or satisfactory. |
|  | **Adoption**; uptake; utilization; initial implementation; intention to try | Adoption is defined as the intention, initial decision, or action to try or employ an innovation or evidence-based practice |
|  | **Appropriateness**; perceived fit; relevance; compatibility; suitability; usefulness; practicability | Appropriateness is the perceived fit, relevance, or compatibility of the innovation or evidence-based practice for a given practice setting, provider, or consumer; and/or perceived fit of the innovation to address a particular issue or problem |
|  | **Feasibility**; actual fit or utility; suitability for everyday use; practicability | Feasibility is defined as the extent to which a new treatment, or an innovation, can be successfully used or carried out within a given agency or setting |
|  | **Fidelity**; delivered as intended; adherence; integrity; quality of program delivery | Fidelity is defined as the degree to which an intervention was implemented as it was prescribed in the original protocol or as it was intended by the program developers |
|  | **Implementation  Cost**; marginal cost; cost-effectiveness; cost-benefit | Cost (incremental or implementation cost) is defined as the cost impact of an implementation effort. Implementation costs vary according to three components. |
|  | **Penetration**; reach; level of institutionalization? Spread? Service access? | Penetration is defined as the integration of a practice within a service setting and its subsystems |
|  | **Sustainability**; maintenance; continuation; durability; incorporation; integration; institutionalization; sustained use; routinization; | Sustainability is defined as the extent to which a newly implemented treatment is maintained or institutionalized within a service setting’s ongoing, stable operations. |
|  | Pain intensity | Impact on how much a patient hurts, reflecting the overall magnitude of the pain experience; NRS 11-point scale |
|  | Physical functioning | Impact on patient’s ability to carry out daily physical activities required to meet basic needs, ranging from self-care to more complex activities that require a combination of skills; FRI |
|  | Quality of life | Impact on physical, psychological and social domains of health, seen as distinct areas that are influenced by a person’s experiences, beliefs, expectations and perceptions; NDI, ODI |
|  | Work disability | Impact on a worker ability to meet physical and/or psychological work demands; FABQ |
|  | Return to work | Economical impact on paid or unpaid job employment due to low back pain, including absenteeism and presenteeism |
|  | Global improvement | CGI/PGI |
|  | Patient satisfaction | Impact on patient’s satisfaction in performing usual social roles and activities (including family and work)  Impact on patient’s satisfaction with care received, including treatment and care providers |
|  | Adverse events | Anticipated and unanticipated adverse events in accordance with CONSORT extension for Better Reporting of Harms |
